# Autosomal-dominant macular dystrophy linked to a chromosome 17 tandem duplication

**DOI:** 10.1172/jci.insight.178768

**Published:** 2024-12-06

**Authors:** Rabiat Adele, Rowaida Hussein, Erika Tavares, Kashif Ahmed, Matteo Di Scipio, Jason Charish, Minggao Liang, Simon Monis, Anupreet Tumber, Xiaoyan Chen, Tara A. Paton, Nicole M. Roslin, Christabel Eileen, Evgueni Ivakine, Nishanth E. Sunny, Michael D. Wilson, Eric Campos, Raju V.S. Rajala, Jason T. Maynes, Philippe P. Monnier, Andrew D. Paterson, Elise Héon, Ajoy Vincent

**Affiliations:** 1 Genetics & Genome Biology program, Hospital for Sick Children (HSC), Toronto, Ontario, Canada.; 2 Vision Division, Krembil Research Institute, Toronto Western Hospital, Toronto, Ontario, Canada.; 3 Department of Molecular Genetics, University of Toronto (U of T), Toronto, Ontario, Canada.; 4 Department of Ophthalmology and Visual Sciences and; 5 The Centre for Applied Genomics, HSC, Toronto, Ontario, Canada.; 6 Department of Animal and Avian Sciences, University of Maryland, College Park, Maryland, USA.; 7 Departments of Ophthalmology, Physiology, and Cell Biology and Dean McGee Eye Institute, University of Oklahoma Health Sciences Center, Oklahoma City, Oklahoma, USA.; 8 Molecular Medicine program and; 9 Department of Anesthesia and Pain Medicine, HSC, Toronto, Ontario, Canada.; 10 Department of Physiology, Faculty of Medicine, and; 11 Department of Ophthalmology and Visual Sciences, U of T, Toronto, Ontario, Canada.

**Keywords:** Genetics, Ophthalmology, Genetic diseases, Genetic variation, Retinopathy

## Abstract

Hereditary macular dystrophies (HMDs) are a genetically diverse group of disorders that cause central vision loss due to photoreceptor and retinal pigment epithelium (RPE) damage. We investigated a family with a presumed novel autosomal-dominant HMD characterized by faint, hypopigmented RPE changes involving the central retina. Genome and RNA sequencing identified the disease-causing variant to be a 560 kb tandem duplication on chromosome 17 [NC_000017.10 (hg19): g.4012590_4573014dup], which led to the formation of a novel *ZZEF1-ALOX15* fusion gene, which upregulates *ALOX15*. *ALOX15* encodes a lipoxygenase involved in polyunsaturated fatty acid metabolism. Functional studies showed retinal disorganization and photoreceptor and RPE damage following electroporation of the chimera transcript in mouse retina. Photoreceptor damage also occurred following electroporation with a native *ALOX15* transcript but not with a near-null *ALOX15* transcript. Affected patients’ lymphoblasts demonstrated lower levels of ALOX15 substrates and an accumulation of neutral lipids. We implicated the fusion gene as the cause of this family’s HMD, due to mislocalization and overexpression of ALOX15, driven by the *ZZEF1* promoter. To our knowledge, this is the first reported instance of a fusion gene leading to HMD or inherited retinal dystrophy, highlighting the need to prioritize duplication analysis in unsolved retinal dystrophies.

## Abstract 2

A fusion gene derived from tandem duplication results in ectopic overexpression of ALOX15 and causes autosomal dominant hereditary macular dystrophy in humans.

## Graphical abstract

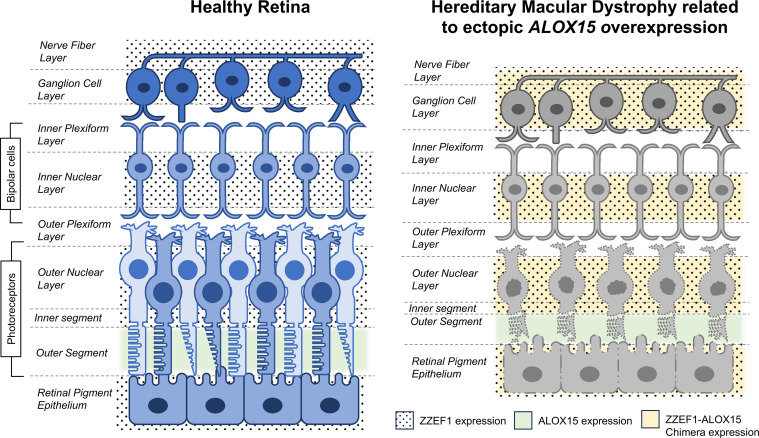

## Introduction

Hereditary macular dystrophies (HMDs) are a group of rare, genetically heterogeneous disorders that affect the central retinal photoreceptors and retinal pigment epithelium (RPE), causing progressive loss of vision (1). HMDs are phenotypically diverse, with some known to have intra- and/or interfamilial variability (1). The disease pathology begins within the macular region but can extend to surrounding retinal regions (2). Variants in over 20 genes have been associated with HMD (3). However, a proportion of HMD subtypes remain uncharacterized, and there are some where the disease-causing genes or variants are yet to be identified (2, 4).

Numerous genetic and biochemical mechanisms underlie HMD pathology, including fatty acid dysregulation. Long chain and very long chain (VLC) polyunsaturated fatty acids (PUFAs) are integral to retinal photoreceptor and pigment epithelial structure, and VLC-PUFAs modulate synaptic transmission (5–7). For example, variants in elongation of very long chain fatty acids 4 (*ELOVL4*), encoding an enzyme involved in the retinal synthesis of VLC-PUFAs (C_28_–C_38_), have been associated with an autosomal-dominant (AD) form of HMD (OMIM: 600110) (8).

Our research implicates another gene involved in fatty acid metabolism, arachidonate 15-lipoxygenase (*ALOX15*), as a presumed novel cause for a rare form of AD HMD. We identified a 560 kb tandem duplication on chromosome (chr) 17 that generates a *ZZEF1*-*ALOX15* fusion gene as the causal variant in a British ancestry family. As a result of the fusion, *ALOX15* is expressed from the *ZZEF1* promoter, resulting in supranormal levels of the enzyme within cells. *ALOX15* encodes 12/15-lipoxygenase, which metabolizes PUFAs — arachidonic acid (AA), docosahexaenoic acid (DHA), eicosapentaenoic acid (EPA), and linoleic acid — to produce bioactive lipid metabolites (e.g., 15-hydroxyeicosatrienoic acid [15-HETE], 12-HETE, lipoxins, hepoxilins, resolvins, protectins) (9, 10). Patient-derived lymphoblast cell lines (LCLs) showed reduced levels of ALOX15 substrates, consistent with an increase in ALOX15 enzyme activity. Retinal electroporation experiments in mice using the chimera construct showed evidence of photoreceptor and RPE damage. To our knowledge, this is the first instance of a fusion gene causing an inherited retinal dystrophy (IRD).

## Results

### Clinical phenotyping reveals a rare HMD.

**Figure 1:**
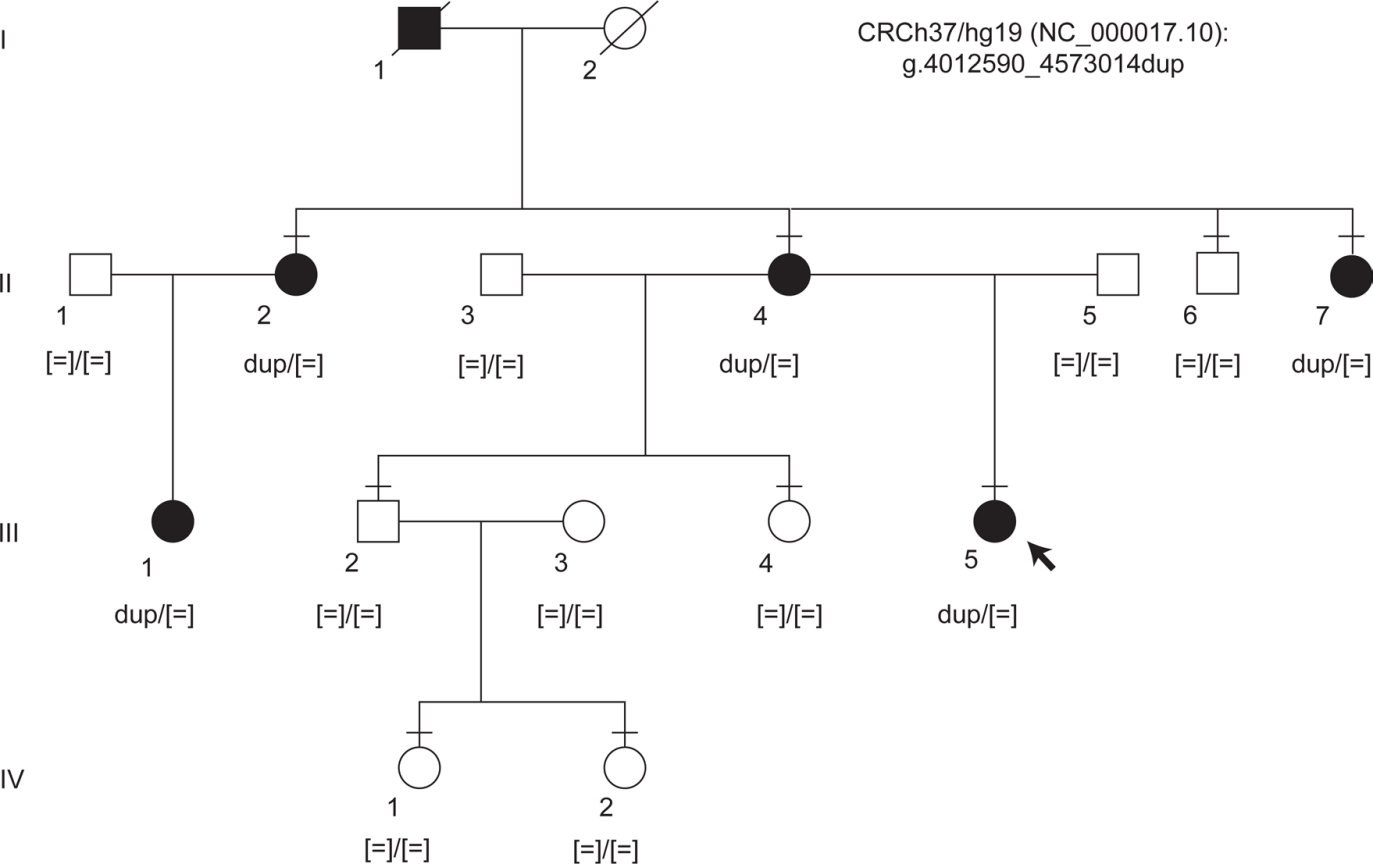
Pedigree structure and segregation results for the chr 17 duplication. Standard pedigree notation was used. A horizontal bar indicates individuals who have been clinically examined. Sanger sequencing confirmed disease segregation with the chr 17 duplication.

**Figure 2:**
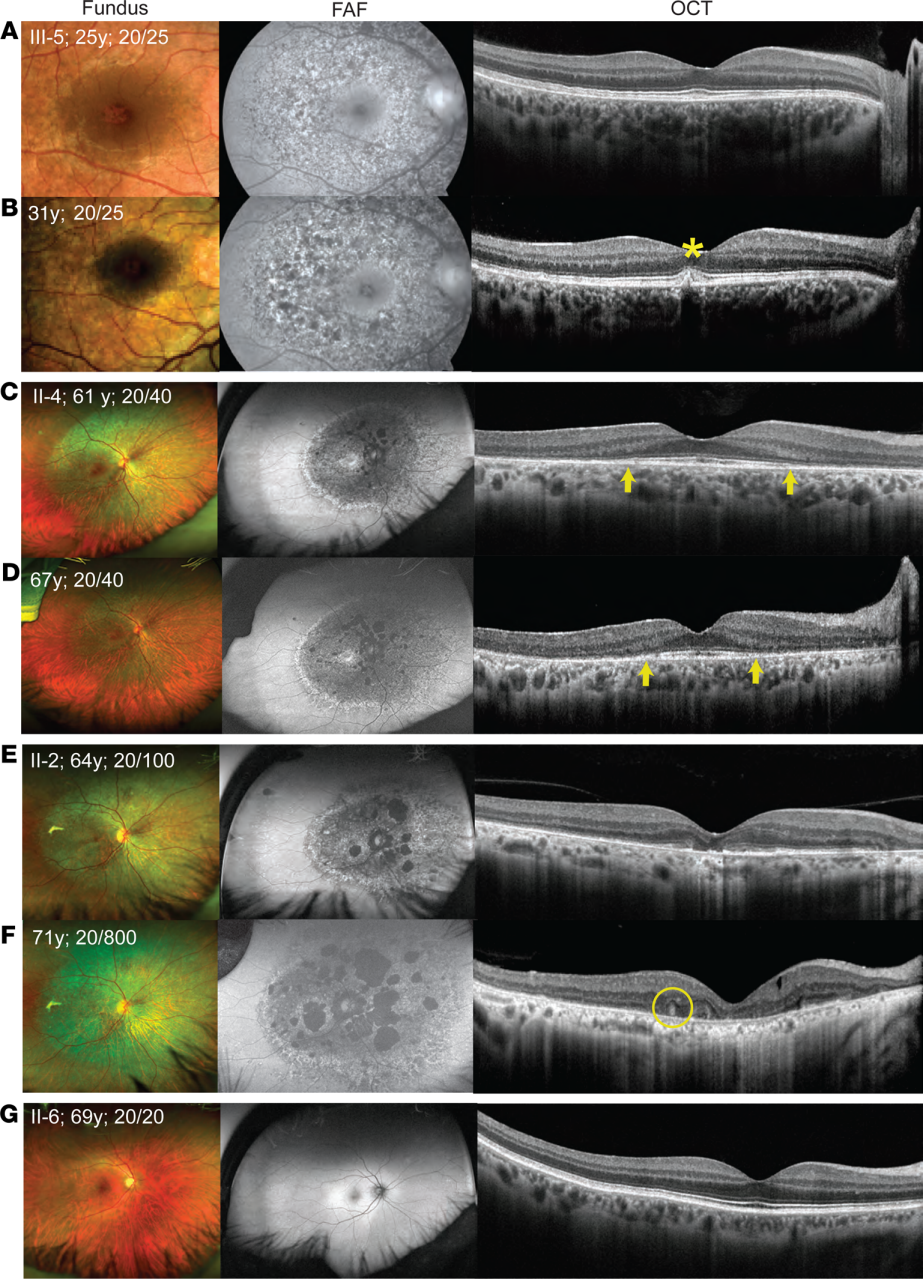
Retinal imaging from the right eye of 1 unaffected and 3 affected family members. Each row has 3 images of an individual from a time point — from left to right these represent color fundus photography, fundus autofluorescence (FAF) imaging, and optical coherence tomography (OCT). (**A** and **B**) Images from proband at 25 and 31 years, respectively. (**C** and **D**) Images from II-4 at 61 and 67 years, respectively. (**E** and **F**) Images from II-2 at 64 and 71 years, respectively. (**G**) Images from an unaffected family member (II-6) at 69 years who serves as a control. The visual acuity (VA) at respective ages is indicated on the fundus image. Fundus shows faint, confluent, deep retinal white dots distributed concentrically at the macula and surrounding regions in all affected individuals, which gradually increased with age. On FAF, these changes resulted in speckled hyper- (increased lipofuscin in retinal pigment epithelium; RPE) and hypo-autofluorescence (RPE atrophy) pattern. The OCT showed evidence of RPE deposition (asterisk), iso-reflective whorl-like structure (circle), and outer retinal damage. There is disruption of the RPE-photoreceptor complex, and the arrows demarcate the extent of preservation of the ellipsoid zone (EZ), which became narrower over time in II-4, suggestive of progressive retinal damage. In II-4 and II-2, marked RPE thinning was also observed.

A family with 6 affected members across 3 generations was recruited (Figure 1). The clinical phenotype is summarized in Figure 2 and Supplemental Table 1; supplemental material available online with this article; https://doi.org/10.1172/jci.insight.178768DS1 Three affected individuals underwent detailed phenotyping (III-5, II-4, and II-2) while 2 affected members only had visual acuity measurements and fundus evaluation (II-7, III-1). I-1 was identified to have HMD based on medical records.

The proband (III-5; Figure 2A), a 24-year-old asymptomatic woman, was referred following an incidental diagnosis of HMD. Retinal examination showed faint, confluent, hypopigmented RPE changes in a concentric manner involving the para- and perifoveal regions, as well as the paramacular regions extending nasal to the disc. The FAF imaging showed a distinct speckled pattern of hyper- and hypo-autofluorescence in the affected regions. OCT showed fine, discrete, hyperreflective deposition at the RPE that led to focal disruptions at the photoreceptor outer segments (POSs) and EZ. Full-field electroretinogram (ERG), pattern ERG, electro-oculogram (EOG), and visual fields were normal (Supplemental Figure 1A and Supplemental Figure 2A).

The proband’s mother (II-4, 61 years, Figure 2C) and one of her aunts (II-2, 64 years, Figure 2E) showed a similar pattern on fundus and FAF imaging but had more evident RPE damage that encroached to the fovea centrally and extended to the midperipheral retina. Their FAF images also demonstrated patchy atrophy. On OCT imaging, II-4 showed complete disruption of the POS and EZ in the peripheral macula with minimal disruption of the central external limiting membrane (ELM). Similar OCT findings were seen in II-2 but with more severe central involvement of the POS, EZ, and ELM. In both II-4 and II-2, the ERG and EOG were normal, but the pattern ERG was abnormal, suggestive of macular dysfunction (Supplemental Figure 1, B and C; Supplemental Figure 1D is a control). The visual fields showed a large, paracentral annular scotoma in II-4 and a large central scotoma in II-2 (Supplemental Figure 2, B and C).

Two affected individuals (III-5 and III-1) had normal distance VA and were asymptomatic at 24 and 18 years of age, respectively. The other affected individuals (II-4, II-2, and II-7) had reduced VA in at least 1 eye (worse than 20/40) at 61, 64, and 60 years of age, respectively. On follow-up exams (6–8 years later), disease progression was observed on FAF and OCT imaging in all 3 patients examined (III-5, II-4, and II-2; Figure 2, B, D, and F). Figure 2G is from an unaffected family member (II-6) who serves as a control.

In summary, the appearance and distribution of the retinal changes suggested a progressive HMD phenotype that we believe is novel.

### Preliminary genetic testing is inconclusive.

Clinical panel-based genetic testing results were inconclusive for II-4 and II-2 (see Supplemental Methods i and Supplemental Table 2). Mitochondrial genome sequencing in III-5 did not identify any pathogenic variants.

### Linkage analysis specifies genomic regions of interest.

Linkage analysis assuming AD inheritance revealed a maximum logarithm of odds (lod) score of 1.79 for 10 regions on 9 autosomes. A lod score of 1.20 was obtained for chromosome X. In total, these 11 regions spanned 119 cM (61.7 Mb), helping narrow our study to 3.3% of the genome (Supplemental Table 3).

### Genome sequencing identifies a tandem duplication on chr 17.

**Figure 3:**
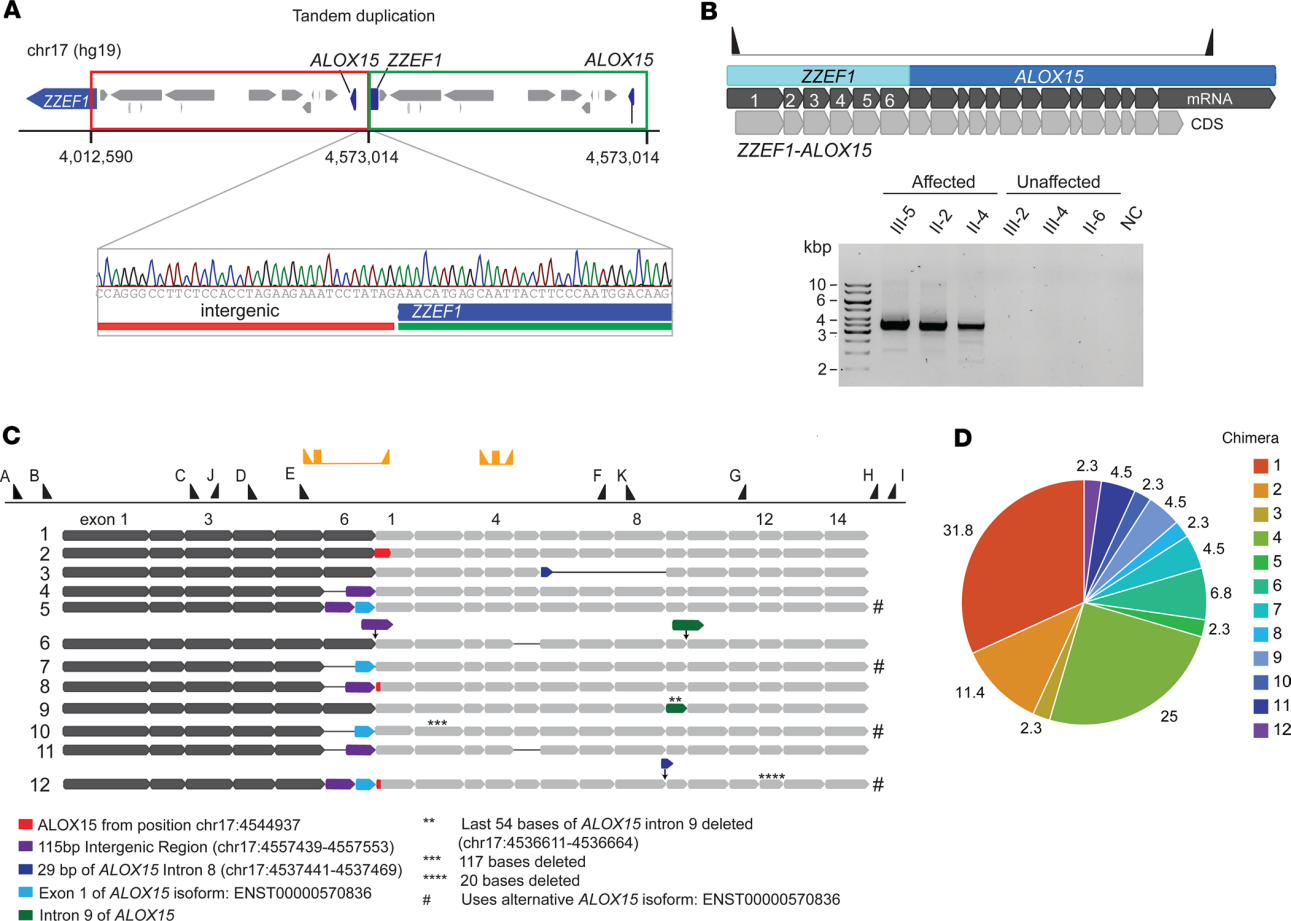
Molecular determination of the fusion gene and chimeric transcripts. (**A**) Schematic of the genic region of chr 17 and sequence view of the junction of the tandem duplication. (**B**) Schematic of the fusion gene and the transcript (top panel) and segregation of the chimeric transcript using gel electrophoresis (bottom panel). Only affected individuals show bands of matching size to the predicted model for the Chimera 1 transcript. NC, negative control. (**C**) Schematic representation of the 12 chimeric transcript isoforms captured by cloning and Sanger sequencing. The dark gray thick arrow bars represent the *ZZEF1* transcript, while the light gray thick arrow bars represent the *ALOX15* transcript. The numbers on exons correspond to the exon number from NM_001140.3 (*ALOX15*), ENST00000570836.1 (alternative *ALOX15* isoform), and NM_015113.3 (*ZZEF1*) transcripts. Hashtag (#) symbols are used to indicate chimeric transcripts of *ALOX15* ENST00000570836.1 isoform. Thick arrow bars in assorted colors represent sections of the transcript different from the Chimera 1 transcript as defined by the legend below the image. Black thin lines between arrow bars represent exon skipping. Thin arrows pointing down represent insertions. Black triangles along the top represent primer positions along the fusion gene used in Sanger sequencing (A=pJET forward primers B=p5516, C=p5670, D=p5581, E=p5518, F=p5582, G=p5673, H=p5578, I=pJET reverse, J=p5772, and K=p5773; Supplemental Table 5). The yellow triangles and rectangles represent chimera and ALOX15 primers and probe for digital PCR. (**D**) Relative frequency of each chimeric transcript found by cloning. A total of 44 CFU were sequenced. Chimera 1 (31.8%) and 4 (25%) are most abundant.

Two affected patients (III-5 and II-2) underwent genome sequencing (GS). Data were filtered to prioritize disease-causing rare candidate variants within the linkage regions that were shared between these 2 individuals, assuming AD inheritance (Supplemental Figure 3). After filtering, 1 structural variant (SV) remained, a 560 kb heterozygous tandem duplication in chr 17 starting in intron 6 of *ZZEF1* and ending in an intergenic region upstream of *ALOX15* [NC_000017.10 (hg19): g.4012590_4573014dup] (Figure 3A). Also, 9 single nucleotide variants (SNVs) and indels remained (Supplemental Figure 3 and Supplemental Table 4). One exonic SNV with low predictive pathogenicity scores was identified in the poly(A) polymerase beta gene [NM_020144.4 (*PAPOLB*): c.1681G>A, p.(Ala561Thr); dbSNP rs528192180] expressed mainly in the testes (11, 12). The remaining SNVs were 5′-UTR, 3′-UTR, intronic, or intergenic variants (Supplemental Table 4), but none had a plausible link to any HMD candidate genes.

The candidate variant most likely to be disease causing was the tandem duplication on chr 17, which was validated and segregated by Sanger sequencing (Figure 3, A and B, and Supplemental Table 5). It comprised 10 coding genes (*ZZEF1*, *CYB5D2*, *ANKFY1*, *UBE2G1*, *SPNS3*, *SPNS2*, *MYBBP1A*, *GGT6*, *SMTNL2*, *ALOX15*), all expressed in the retina (13). *ANKFY1*, *SPNS2*, and *ALOX15* had possible implications for an eye phenotype, based on existing literature (10, 14, 15). This duplication was absent from public population databases for copy number variants (CNVs) — DGV (16) and gnomAD (SVs) 4.0 (17) — and from publications based on CNV or whole exome data from the UK Biobank (18–21).

### RNA sequencing reveals overexpression of ALOX15 in patient lymphoblasts.

**Figure 4:**
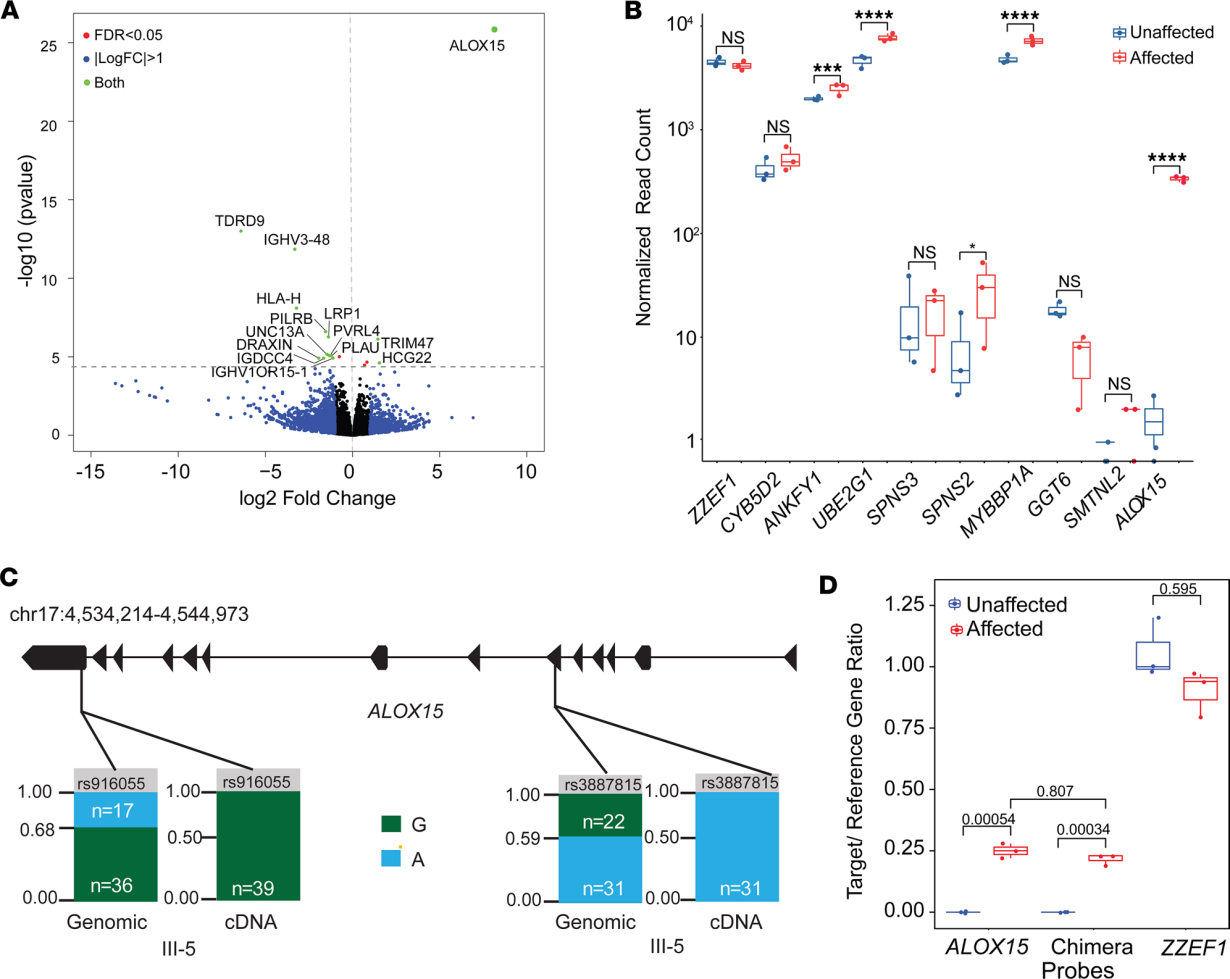
RNA DGE analysis. (**A**) Volcano plot showing differentially expressed genes (DEGs) in affected (*n* = 3) and unaffected LCLs (*n* = 3). The *x* and *y* axes indicate the log_2_fold-change of genes and corrected Benjamini-Hochberg *P* value, respectively. Genes above the horizontal blue line are differentially expressed with a *P* < 0.05. Blue dots represent genes with a log_2_fold-change > 1, the red dots indicate DEGs with a *P* < 0.05, and the green dots are genes that have both a significant *P* value and fold-change. Black dots neither have a significant *P* value nor have a significant fold-change. Genes to the left of the vertical line are underexpressed, while genes to the right are overexpressed, in affected compared with controls. *ALOX15* is highly upregulated in patients. (**B**) Box plot graph comparing the expression of genes within the chr 17 duplication between the 3 unaffected and 3 affected family members. *ALOX15* had the greatest difference in gene expression (**** = *P* ≤ 0.0001, *** = *P* ≤ 0.001, * = *P* ≤ 0.05) (unpaired, 2-sample *t* test). Box plots show the interquartile range, median (line), and minimum and maximum (whiskers). (**C**) *ALOX15* allele count comparison in genomic data versus RNA-Seq data in the proband. Bar charts display the allele read count of the G allele (green) and the A allele (blue) at each position. Both single nucleotide polymorphisms showed preferential expression for an allele at the RNA level. (**D**) Comparison of the expression levels of 3 transcripts (Chimera, *ALOX15*, and *ZZEF1*), between affected (*n* = 3) and unaffected family members (*n* = 3), using droplet digital PCR. The *y* axis shows the normalized target gene expression. Only the affected express the chimeric transcript and *ALOX15*. For *ZZEF1*, the expression levels between the 2 groups are similar (2-sample *t* test, Bonferroni-adjusted *P* values indicated on the graph).

RNA sequencing (RNA-Seq) was performed using LCLs from 3 affected and 3 unaffected family members, and the reads were aligned to the hg19 reference genome (Supplemental Table 6). Differential gene expression (DGE) analysis revealed 14 genes significantly differentially expressed (3 upregulated and 11 downregulated; corrected *P* < 0.05) (Figure 4A and Supplemental Table 7). Only 3 of the differentially expressed genes, *HCG22*, *ALOX15* (both upregulated in patients), and *HLA-H* (downregulated in patients) were within the linkage regions. The most differentially expressed gene was *ALOX15*, which showed an over 200-fold change (adjusted *P* = 3.12 × 10^–22^; Figure 4A and Supplemental Table 7). *ALOX15* encodes 12/15-lipoxygenase, involved in PUFA metabolism (Supplemental Figure 4).

Gene expression differences between the unaffected and affected family members for the 10 genes within the duplication were assessed (Figure 4B). Four genes had a significant fold-change of approximately 1.5, as expected for a tandem duplication: *ANKFY1* (*P* = 0.00064), *UBE2G1* (*P* = 0.000022), *MYBBP1A* (*P* = 0.000035), and *SPNS2* (*P* = 0.036). Five other genes did not show a significant fold-change: *ZZEF1* (*P* = 0.97), *CYB5D2* (*P* = 0.086), *SPNS3* (*P* = 0.86), *GGT6* (*P* = 0.21), and *SMTNL2* (*P* = 0.46). No obvious relationship was observed between genomic position and DGE on chr 17 between affected and unaffected individuals (Supplemental Figure 5). The only Bonferroni-significant DGE on chr 17 was *ALOX15* (*P* < 0.00001).

### ZZEF1-ALOX15 chimeric transcript was identified in patient lymphoblasts.

Split reads in regions of *ALOX15* that mapped to *ZZEF1* and vice versa were observed in RNA-Seq data from patients but not in unaffected family members. Both genes are encoded on the reverse strand, and the tandem duplication positions exons 1 to 6 of *ZZEF1* upstream of *ALOX15*. This results in patients expressing a chimeric *ZZEF1-ALOX15* transcript under the regulation of the *ZZEF1* promoter. RNA-Seq reads (from III-5) were then mapped to the predicted chimera model (Figure 3B), where numerous reads mapped to the model in the junction region. This transcript is referred to as the *ZZEF1-ALOX15* Chimera 1 (Chimera 1). Gel electrophoresis performed on cDNA verified the presence of the chimeric transcript only in affected patients (~3,500 bp, matching predicted size) (Figure 3B), as confirmed by Sanger sequencing.

Variations of the chimeric *ZZEF1-ALOX15* transcript were also detected. PCR cloning and Sanger sequencing of cDNA from the proband identified 11 additional chimeric transcripts (Figure 3C). These transcripts were similar to Chimera 1 but had alterations such as skipping of exon 6 of *ZZEF1*, insertion of intergenic regions between truncated *ZZEF1* and *ALOX15*, insertion of *ALOX15* introns, base deletions, and exon skipping in *ALOX15*. Most transcripts (Chimera 1, 2, 3, 4, 6, 8, 9, 11) matched the main *ALOX15* transcript (NM_001140.3), whereas 4 transcripts (Chimera 5, 7, 10, and 12) matched an alternative isoform of *ALOX15* (ENST00000570836.1). Chimera 1 (31.8%) and 4 (25%) were the most abundant (Figure 3D). Chimera 1, 2, 3, 4, 7, 8, 9, and 10 were in frame.

### The duplicated allele is selectively overexpressed in affected patients.

To assess whether the overexpression of *ALOX15* seen in affected individuals originated from the duplicated or nonduplicated allele, we used 2 exonic heterozygous single nucleotide polymorphisms (SNPs) on *ALOX15* [rs916055 (A>G) and rs3887815 (G>A)] found on GS data (III-5; Supplemental Methods xi). Both SNPs had an allele ratio of approximately 1:2 in GS, consistent with a heterozygous duplication (Figure 4C). From the allele ratios it was inferred that G was on the duplicated allele for rs916055 and A was on the duplicated allele for rs3887815. RNA-Seq data from patient samples demonstrated only the G and A allele expression, respectively. This suggested that only the duplicated allele that generates the chimeric transcript is expressed in affected patients.

### ALOX15 overexpression is driven by ZZEF1 promoter in patient lymphoblasts.

Droplet digital PCR (ddPCR) results from LCLs verified that affected individuals expressed the *ZZEF1*-*ALOX15* chimeric transcript 59-fold higher than unaffected individuals (*P* = 0.000116) and the *ALOX15* transcript over 220-fold compared with unaffected individuals (*P* = 0.000099; Figure 4D). Furthermore, in affected individuals, the levels of the chimera and *ALOX15* transcripts were similar (Figure 4D), suggesting that most of *ALOX15*’s expression is driven by the *ZZEF1* promoter. The median expression of *ZZEF1* was comparable between affected and unaffected individuals (*P* = 0.15).

### ZZEF1-ALOX15 chimera protein is detected in patient lymphoblasts.

**Figure 5:**
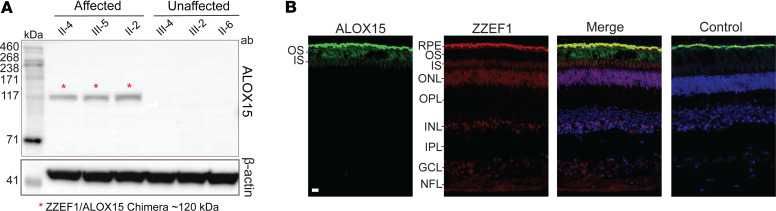
ALOX15 protein analysis in patient-derived lymphoblast cells and human retina. (**A**) Western blot showing ZZEF1-ALOX15 chimera protein expression in patients. Immunoreactive bands with a molecular weight of ~120 kDa were observed only in the affected patients’ lanes, which corresponded to the predicted chimera molecular weight. The membrane section underneath was probed with β-actin antibody, as a loading control. (**B**) ALOX15 and ZZEF1 expression in human retinal cryosections. ALOX15 (green) is expressed in photoreceptor outer segments (OS). ZZEF1 (red) shows expression in the inner segments (IS), outer nuclear layer (ONL), inner nuclear layer (INL), ganglion cell layer (GCL), and nerve fiber layer (NFL). DAPI is stained in blue. Control was incubated with secondary antibodies only. The bright signal in the retinal pigment epithelium (RPE) is nonspecific as it is present in all channels in the negative control. Scale bar: 20 μm.

Western blot results showed that only affected members had a band at approximately 120 kDa (detected by ALOX15 antibody; Supplemental Methods xiii), matching the size predicted for the chimera transcript (Figure 5A). No bands were observed at the predicted molecular size of ALOX15 (~75 kDa) in either affected or unaffected family members. This was expected as endogenous levels of *ALOX15* are extremely low, as shown in the RNA-Seq data.

### ZZEF1 and ALOX15 have distinct expression patterns in normal human retina.

Immunostaining from a control human donor eye revealed ALOX15 expression predominantly restricted to the POS (Figure 5B; Supplemental Methods xiv). Comparatively, ZZEF1 had a broader expression pattern, with signal visible in the outer nuclear layer (ONL), the nerve fiber layer (NFL), the ganglion cell layer (GCL), a subset of cells in the inner nuclear layer (INL), and the inner segment (IS) (Figure 5B). The bright signal evident in the RPE is due to nonspecific binding from secondary antibody. In literature, ZZEF1 but not ALOX15, is found to be expressed in hTERT-RPE1 cells, a model for the RPE (22).

### ALOX15 substrates are reduced in patient lymphoblasts.

**Figure 6:**
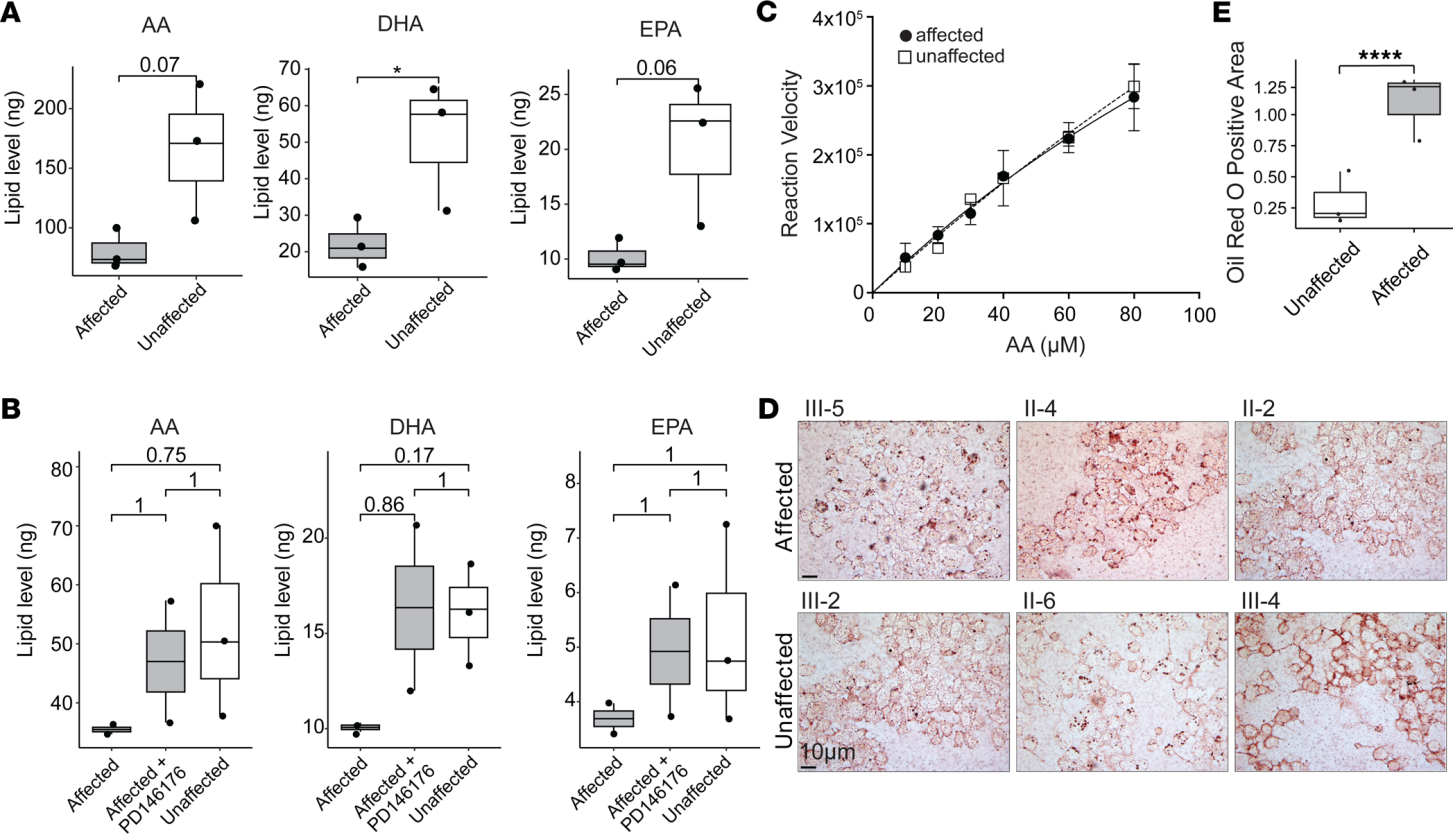
Analysis of lipid profile in affected individuals. (**A**) Box plots showing PUFA substrate levels from LCLs in 3 affected and 3 unaffected individuals. Affected individuals demonstrated lower levels of arachidonic acid (AA) (*P* = 0.07), docosahexaenoic acid (DHA) (*P = 0.05), and eicosapentaenoic acid (EPA) (*P* = 0.06) (2-tailed, unpaired *t* test). Box plots show the interquartile range, median (line), and minimum and maximum (whiskers). (**B**) Following treatment with PD146176, an inhibitor of ALOX15, the substrate levels were restored in affected individuals (Bonferroni-corrected *P* values, 2-tailed, unpaired *t* test). (**C**) Michaelis-Menten curve plotting AA concentration (μM) on the *x* axis and reaction velocity (product formation/time) on the *y* axis. Both chimeric and WT enzymes showed similar substrate affinity (*K_m_* of 6.4 and 8.4 μM, respectively). LCLs from II-4 and II-6 were used in this assay. (**D** and **E**) Oil Red O analysis indicated significantly higher levels of neutral lipids in LCLs derived from affected individuals (*n* = 3) compared with unaffected (*n* = 3; ****P < 0.0001; 2-tailed Mann-Whitney test). Images taken at 63× original magnification with oil immersion. Scale bar: 10 μm.

Liquid chromatography-mass spectrometry (LC-MS) revealed affected patient LCLs (*n* = 3) to have lower mean levels (ng/μL) of AA (affected: 80.8 ± 17.8 [SD]; unaffected: 166.2 ± 56.2; *P* = 0.07), DHA (affected: 21.8 ± 6.6; unaffected: 51.4 ± 17.9; *P* = 0.05), and EPA (affected: 10.2 ± 1.5; unaffected: 20.3 ± 6.7; *P* = 0.06) compared with unaffected family members (*n* = 3) (Figure 6A).

In a separate experiment, when patient LCLs were treated with PD146176, an inhibitor of ALOX15, a recovery in AA (affected: before treatment: 35.47 ± 1.07 after treatment: 47.01 ± 14.61; unaffected: 52.76 ± 16.25), DHA (affected: before treatment: 10.07 ± 0.33 after treatment: 16.35 ± 6.16; unaffected: 16.04 ± 2.64), and EPA (affected: before treatment: 3.69 ± 0.40 after treatment: 4.92 ± 1.69; unaffected: 5.22 ± 1.83) levels was noted in affected individuals (Figure 6B). These results verify the chimeric enzyme activity to account for the lower substrate levels in the patient LCLs as opposed to inherently low PUFAs within the cell lines.

### Chimeric and WT enzymes have similar substrate affinity.

We performed an enzyme kinetics assay by plotting reaction velocity against substrate concentration to generate a Michaelis-Menten curve. The results showed the ALOX15 substrate affinity to be similar in affected and unaffected individuals (Michaelis constant, *K_m_*, of 6.4 and 8.4 μM, respectively; Figure 6C). Further, the 15-HETE product formation rate was indistinguishable between affected and unaffected LCLs.

### ALOX15 metabolites are low in patient and unaffected lymphoblasts.

The levels of AA (15- and 12-HETE), DHA (14-hydroxy DHA and 17S-hydroxy DHA), and EPA-derived (15S-hydoxy EPA and 12S-hydoxy EPA) metabolites in patients (*n* = 3) and unaffected LCLs (*n* = 3) were at the lower end of the LC-MS detection curve (Supplemental Figure 6, A, D, and E). These low values were susceptible to variability as seen in the 15- and 12-HETE levels on repeated testing (Supplemental Figure 6, B and C). Further downstream metabolites of AA and DHA, such as lipoxins, resolvins, and protectins, were nondetectable. The lack of higher levels of metabolites in patient LCLs may be due to rapid clearance to the cell media.

### Increased ALOX15 activity results in elevated neutral lipids in patient LCLs.

Since ALOX15 is involved in lipid metabolism (Supplemental Figures 4 and 7), we stained LCLs with Oil Red O (ORO) to quantify neutral lipid levels. Affected patients (*n* = 3) had increased ORO staining compared with unaffected family members (*n* = 3; *P* < 0.0001) (Figure 6, D and E).

### Chimeric and native ALOX15 transcripts damage electroporated mouse retinas.

To explore the in vivo effects of both the chimeric and native *ALOX15* transcripts in photoreceptors, electroporation was performed targeting mitotic progenitor retinal cells of P0 or P1 mice. In the chimera and green fluorescent protein (GFP) control electroporated regions, TUNEL-positive cells were largely absent on P3, indicating no immediate cell death (Supplemental Figure 8).

**Figure 7:**
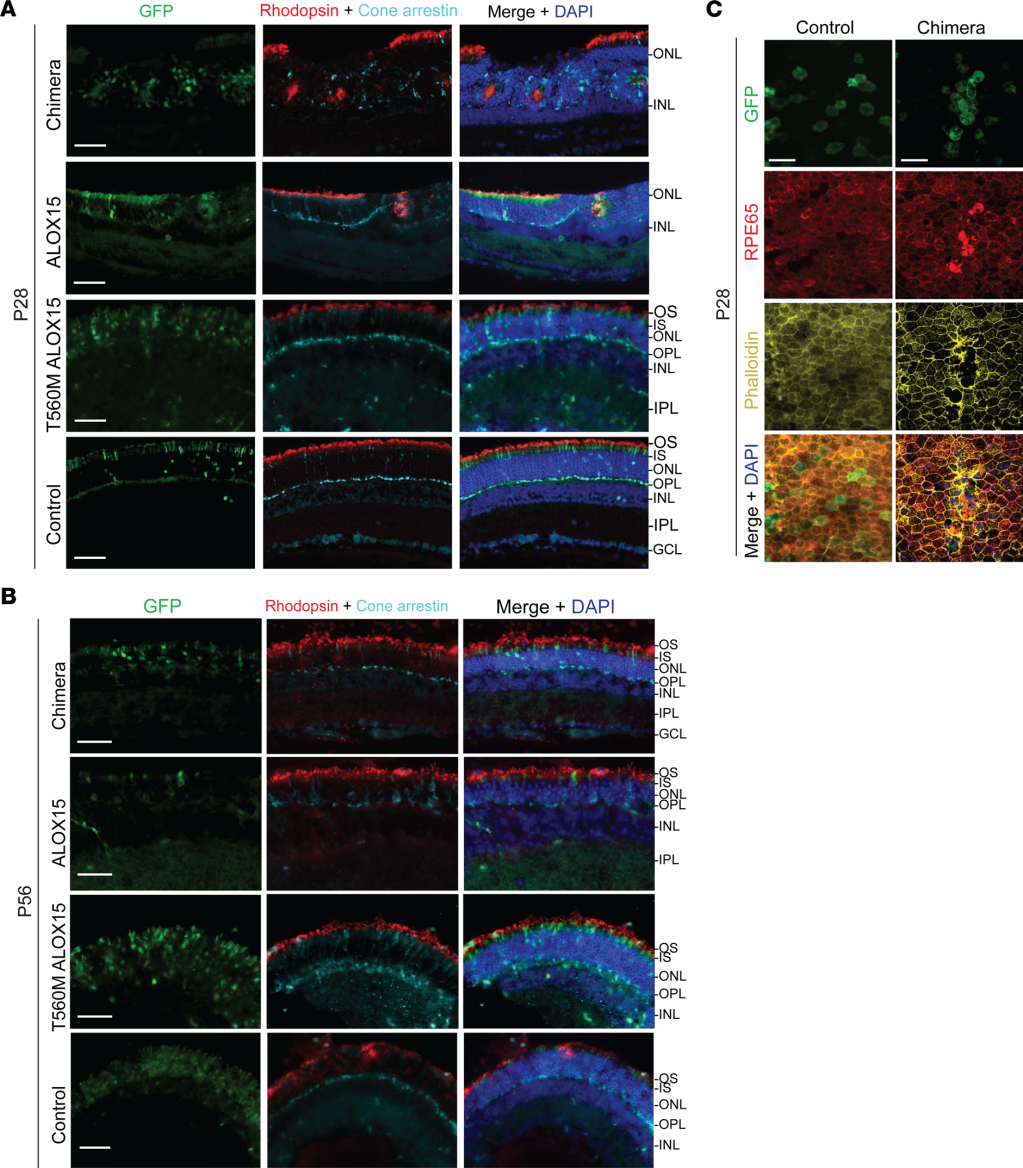
Expression of chimeric and *ALOX15* transcripts in mouse retinas. (**A** and **B**) P0 or P1 C57BL/6J mice were given subretinal injections of pT2K-IRES-eGFP (GFP control), pT2K-chimera-IRES-eGFP (chimera), pT2K-ALOX15-IRES-eGFP (*ALOX15*), or pT2K-CAGGS-IRES-eGFP-*ALOX15*-Thr560Met (T560M *ALOX15*) followed by electroporation of developing photoreceptors. Electroporated cells are labeled in green (GFP). Nuclei were labeled with DAPI (blue), rod outer segments were labeled with rhodopsin (red), and cones were labeled with cone arrestin (cyan). (**A**) At 28 days following electroporation, areas of the outer nuclear layer (ONL) that were electroporated with the chimera (top row) or *ALOX15* (second row) constructs had a high incidence of rosette formation containing both cone and rod cells. Mice electroporated with both the T560M *ALOX15* (third row) and control GFP (bottom row) constructs appeared normal. Three to 4 mice were analyzed in each construct group. (**B**) At 56 days following electroporation, areas of the ONL electroporated with the chimera (top row) or *ALOX15* (second row) constructs appeared thinner than retinas electroporated with T560M *ALOX15* (third row) and control GFP (bottom row). One to 3 mice were analyzed in each construct group. OS, outer segments; IS, inner segments; OPL, outer plexiform layer; INL, inner nuclear layer; IPL, inner plexiform layer; GCL, ganglion cell layer. (**C**) P0 or P1 C57BL/6J mice were given subretinal injections of the chimera plasmid followed by electroporation of RPE. Nuclei were labeled with DAPI (blue), RPE cells were labeled with RPE65 (red), and actin cytoskeleton was labeled with phalloidin (yellow). Compared with GFP-electroporated RPE (left column), RPE electroporated with the chimera show abnormal morphology and exhibit RPE65 upregulation and phalloidin disorganization (right column). These features are consistent with severe RPE damage. Three to 5 mice were analyzed in each group. Images were taken at 20× original magnification. Scale bars: 50 μm.

Cryosections from P28 and P56 mice were examined for retinal abnormalities. At P28, rosette formations within the ONL were observed in retina electroporated with either the chimera or *ALOX15* constructs but not in GFP controls. Further staining with rhodopsin and cone arrestin antibodies showed that the rosettes were made up of rod and cone photoreceptors, respectively (Figure 7A). However, at P56, rosettes were no longer observed, but distinct thinning of the ONL was present in the chimera construct– and *ALOX15* construct–electroporated regions, as opposed to GFP control, which appeared normal (Figure 7B). Further, when electroporated with a construct containing a near-null enzyme (Thr560Met *ALOX15*), cryosections from P28 and P56 mouse retina demonstrated no evidence of rosette formation or ONL thinning, respectively. These findings collectively suggest that the chimera causes retinal photoreceptor damage due to ALOX15 enzymatic activity.

Additionally, we electroporated the RPE of P0 or P1 mice with the chimera construct to observe how chimera expression affects RPE cells. Normally, RPE cells are uniformly sized and exhibit a hexagonal shape, as seen in the GFP-electroporated control (Figure 7C). However, electroporation with the chimera plasmid showed RPE cell damage at P28 as noted by large, misshapen cells that overexpressed RPE65 with associated phalloidin disorganization and stress fiber formation (Figure 7C) (23).

### Patient rephenotyping reveals retinal features resembling electroporation in mice.

All the retinal OCT scans from the macula were reexamined in the 3 affected individuals (III-5, II-2, and II-4) to ascertain for rosettes, ONL thinning, or RPE thinning. Rounded structures were found in II-2 (Figure 2F and Supplemental Figure 9), and ONL and RPE thinning was noted in II-2 (Figure 2, E and F, and Supplemental Figure 9) and II-4 (Figure 2, C and D).

As elevation in ALOX15 or its metabolites has been associated with increased risk of atherogenesis, metabolic dysfunction–associated steatotic liver disease, diabetes, stroke, Alzheimer’s disease, and breast and prostate cancer, detailed rephenotyping was performed (10, 24–30). The only relevant positive history was the presence of invasive ductal carcinoma of the breast in II-2 (at 49 years of age) and symptoms suggestive of Alzheimer’s disease in I-1 (at 62 years of age). The proband and affected mother had normal hemoglobin, HbA1C, peripheral smear, differential count, liver function (and ultrasound), and platelet aggregation (Supplemental Table 8). The proband had a normal lipid profile, while II-4 and II-2 had borderline-high and high cholesterol levels, respectively; neither were on relevant medical treatment.

### Tandem duplication alters topologically associating domain architecture.

**Figure 8:**
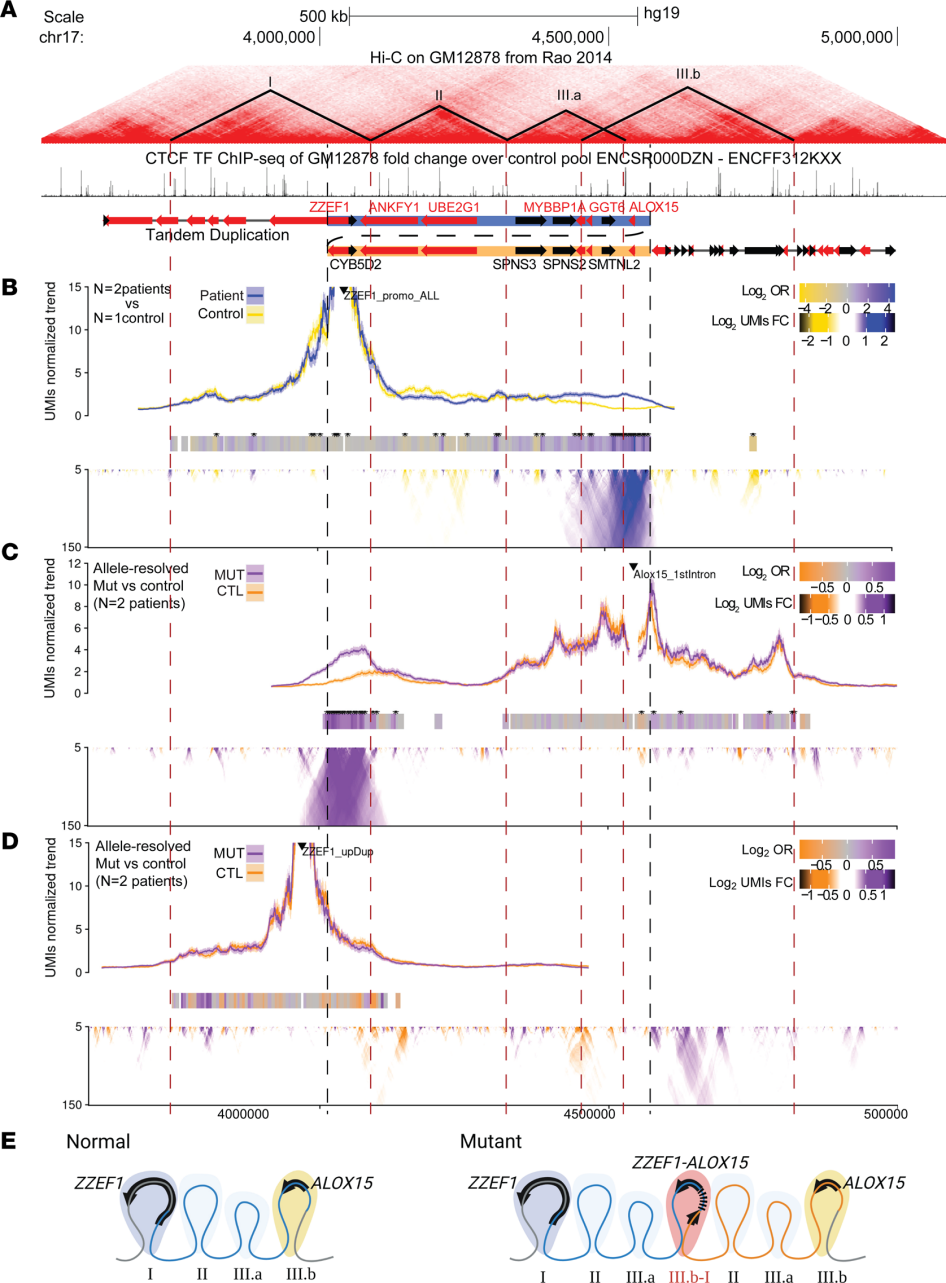
TAD architecture due to the tandem duplication. (**A**) Genome topology around the tandem duplication showing Hi-C contacts, putative TAD boundaries, CTCF binding sites, and genes (32). Black and red arrows denote forward and reverse strand, respectively. Dashed red lines denote TAD boundaries; dashed black lines denote the tandem duplication boundaries. (**B**–**D**) Chromatin contacts were assessed using UMI-4C. Black triangle marks position of baits. Trend lines show the normalized contact frequency. Domainograms show the differential contact frequencies. Bar heatmap with * (adjusted *P* < 0.001) shows regions that are significantly different in contact frequency. Comparisons were made between either the WT and tandem duplicated (Dup) allele in patient samples or between control (*n* = 1) and patient (*n* = 2) samples. (**B**) *ZZEF1* promoter: non-allele-specific bait upstream of the *ZZEF1* promoter shows patient-specific contacts with ALOX15. (**C**) *ALOX15* 1st Intron: allele-specific bait within the first intron of *ALOX15* shows contacts with *ZZEF1* only on the Dup allele. (**D**) *ZZEF1* Dup: allele-specific bait within *ZZEF1* gene body outside of the duplication shows no change in contacts between Dup and WT allele. (**E**) Schematic depicting the “neo-TAD” formed at the duplication junction.

We investigated how the duplication affected the chromatin architecture at the locus. For example, duplications have the potential to generate new long-range interactions that result in the ectopic use of cis-regulatory elements leading to aberrant gene expression (31). Using public Hi-C data from a common LCL cell line (GM12878 cells, Coriell Institute) (32), we identified topologically associating domains (TADs: regions more likely to engage in DNA-DNA interactions) (33) at the locus by inspection of contact matrices and CCCTC-binding factor (CTCF) binding. We displayed these regions in a genome browser view alongside CTCF ChIP-Seq data collected from LCLs (34), which often demarcate the TAD boundary regions (Figure 8A).

In WT LCLs, *ZZEF1* is found in its own TAD (TAD I), and *ANKFY1* and *UBE2G1* are found distally (TAD II), followed by the *ALOX15-*containing TAD (TAD III.a and III.b). This DNA topology suggests that *ZZEF1* and *ALOX15* are normally expressed using distinct *cis*-regulatory elements (Figure 8A). To understand how the tandem duplication impacted chromatin topology at the locus, we generated chromatin conformation capture profiles for regions within and outside of the tandem duplication using patient- (*n* = 2) and control- (*n* = 1) derived LCLs using unique molecular identifier (UMI-4C) approach (35). We found that chromatin contacts from the viewpoint of the *ZZEF1* promoter showed strong chromatin contacts with the *ALOX15* locus only in the patient LCLs (Figure 8B). This observation is consistent with detecting proximal DNA interactions between *ZZEF1* and *ALOX15* because of the tandem duplication. When we used the first intron of *ALOX15* as a reciprocal viewpoint, we were able to use a SNP (rs11568078 [T/C]) that allowed us to distinguish which 4C-Seq signal came from the duplicated allele in each patient (Figure 8C). Consistent with the *ZZEF1* viewpoint, we observed interactions between the *ALOX15* intron viewpoint and the *ZZEF1* gene.

To evaluate whether the tandem duplication altered the chromatin conformation of the *ZZEF1* gene body region (TAD I), we performed 4C-Seq using a viewpoint within the *ZZEF1* gene body proximal to the duplication (Figure 8D). Using a SNP (rs78220873 [T/C]), we compared 4C-Seq signal between the duplicated and WT alleles within each patient. The observed 4C-Seq signal recapitulated the TAD I boundaries, and we found no overt differences between the duplicated and WT alleles. Together, these results support a model whereby the duplication leads to a fusion gene (and a “neo-TAD”) while preserving the neighboring DNA topology (Figure 8E).

## Discussion

We identified a tandem duplication on chr 17 that generates a fusion gene between *ZZEF1* (first 5 or 6 exons) and full-length *ALOX15*, resulting in a rare HMD phenotype. Younger patients with this phenotype showed RPE changes, while the older affected individuals also showed progressive photoreceptor damage and vision loss. Patients’ LCLs aberrantly overexpressed *ALOX15*, driven by the *ZZEF1* promoter, resulting in reduced substrates. Signs of photoreceptor damage were present in mouse retinas electroporated with the chimeric and native *ALOX15* transcripts but not in those electroporated with the near-null *ALOX15* transcript, verifying the deleterious effect of ALOX15 activity. Furthermore, RPE damage occurred following chimera electroporation. Normally, ALOX15 and ZZEF1 are expressed in distinct human retinal layers, and the latter has a more generalized expression pattern. We hypothesize that in affected patients, the chimeric transcript is aberrantly expressed in multiple retinal layers, resulting in HMD. To our knowledge, this is the first report of a chimeric transcript causing HMD and the first to implicate *ALOX15* in HMD, adding new insights to the field.

### Chimera’s functionality based on protein domains and enzyme assay.

The chimeric protein includes all domains of ALOX15 and only the N-terminal portion of ZZEF1 (corresponding to the first 355 [exons 1 to 5]or 425 amino acids [exons 1 to 6] out of 2,961 amino acids [56 exons]), devoid of its 2 main functional domains (ZZ-type zinc finger domains). The function of the first ZZ domain remains unknown, but the second ZZ domain is predicted to act as a histone reader, recognizing and binding epigenetic sites on the N-terminus of histone H3 (36). The absence of ZZ domains and the presence of ALOX15 domains suggests that the chimeric transcript takes on ALOX15 activity confirmed by enzymatic assay. Furthermore, the chimeric and WT enzymes had similar *K_m_* values to those reported in literature (37).

### The ZZEF1 promoter is expected to upregulate ALOX15 expression in multiple tissues.

We validated the predicted structure of the chimeric transcripts; starting at exon 1 of *ZZEF1*, they included the first 5 or 6 exons of *ZZEF1* followed by *ALOX15* in its entirety, and most were in frame. Further, *ALOX15* overexpression was found to be associated only with the duplicated allele, and the levels of expression of the chimeric transcripts were similar to those of WT *ALOX15*. Affected individuals overexpressed *ALOX15* many folds (60 to over 200 times), much higher than the expected 1.5-fold change of a duplicated allele. In contrast, unaffected family members had only residual levels of *ALOX15* transcript, consistent with low endogenous *ALOX15* expression in most tissues (12).

Further, the tandem duplication resulted in a neo-TAD, which contains the *ZZEF1*-*ALOX15* fusion gene but otherwise did not impact chromatin topology at the locus. Based on this result and the gene expression data collected, we suggest that cis-regulatory elements in the *ZZEF1* upstream region through to intron 6 (in TAD I) are responsible for driving the expression of the *ZZEF1*-*ALOX15* fusion gene and *ZZEF1* itself.

As ZZEF1 has a much higher and broader retinal expression pattern compared with ALOX15 (13), we predict the chimeric transcript to induce an ectopic overexpression of ALOX15 in multiple retinal layers. Additionally, single-cell transcriptome data show cone cells to have high *ZZEF1* expression (38). This suggests that the HMD phenotype results from a combination of the *ZZEF1* promoter–driven overexpression of *ALOX15*, and aberrant retinal layer expression of *ALOX15*, particularly in cone photoreceptors, which are most abundant in the macula.

Overall, ZZEF1 is expressed in more tissues than ALOX15 (12, 22, 39, 40). Elevated levels of ALOX15 and its resulting metabolites have been associated with breast cancer and Alzheimer’s disease (28, 29, 41). It is unclear if the overexpression of ALOX15 is associated with any extraretinal phenotype observed in this family, as none was shared among all affected individuals. However, both diseases can also be triggered by multiple other variants or environmental factors (42, 43).

### Role of ALOX15 in humans and mouse models.

Electroporation of the chimeric or native *ALOX15* transcripts showed photoreceptor damage in mouse retina. Additionally, electroporation with the chimera transcript showed RPE cell damage. To our knowledge, no human Mendelian disorder has been previously associated with genetic variations involving *ALOX15*. However, studies have reported damaging and protective effects of over- and underexpressing ALOX15 and its metabolites, in various cell types, mouse models, and human diseases (Supplemental Table 9) (41). For instance, elevated 12-HETE levels have been implicated in dorsal root ganglion axon degeneration (44). Also, overexpression of ALOX15 and its metabolites (12-HETE and 15-HETE) has been implicated in diabetic retinopathy (45, 46). These studies support our electroporation experiments, showcasing *ALOX15*’s capability to cause damaging effects to neurons when overexpressed.

On the contrary, DHA-derived ALOX15 metabolites, such as neuroprotection D1, serve a protective role in the retina (47). Also, overexpression of AA-derived lipoxins (LXA4 and LXB4) was found to be neuroprotective (48). Both LXA4 and LXB4 are metabolites of *ALOX5* (41), an enzyme within the lipoxygenase family, but their synthesis relies on the *ALOX15*-derived precursor 15-HPETE (hydroperoxyeicosatetraenoic acid; Supplemental Figure 4). RNA-Seq data did not show differential expression of *ALOX5* in affected family members (Supplemental Table 10); thus, the ALOX15-ALOX5 circuit is likely not upregulated in this family.

The conflicting evidence regarding the damaging or protective effects of overexpressing ALOX15 in the retina can be attributed to the variety of lipid metabolites derived from its enzymatic activity that have antagonistic functions (9, 10, 41). Further, ALOX15 enzyme activity can be tissue, cell, or disease specific, and its response and targets may vary based on the cellular context, substrate availability, or presence of other regulators or coexpressed enzymes (9, 10, 41, 49). Also, the dosage levels of a bioactive metabolite may play a role; low doses of LXA4 are antiinflammatory, while higher doses can trigger inflammation (50).

Increased ALOX15 activity led to significantly reduced DHA levels in patient LCLs. DHA is the most abundant PUFA in the POS, comprising nearly 50%–60% of phospholipid side chains, and is essential in phototransduction and POS renewal (51). DHA abundance is correlated with retinal protection, while insufficient intake of DHA causes retinal impairment in both humans and animal models (52–54). Hence, reduced DHA levels in the retina due to elevated ALOX15 activity can contribute to photoreceptor damage in our patients.

In addition to DHA, patient LCLs also showed lower levels of AA and EPA. Given that the chimeric enzyme has comparable substrate affinity to WT, our results suggest increased PUFA catabolism by ALOX15, generating bioactive lipids that are likely immediately transported out of the cell. Although the LCL is an easily accessible tissue and patient-derived disease model, it may not represent retinal cell biology.

### A presumed novel HMD phenotype.

The described HMD phenotype predominantly affected the central retina and extended outward. The speckled pattern on FAF imaging that corresponded to the hypopigmented retinal changes suggested RPE dysfunction. The OCT in the young proband (III-5) showed RPE deposition, consistent with this finding. In older affected individuals, the RPE is markedly thinned, suggesting progressive RPE damage. Scalloped retinal atrophy and outer retinal disruption found in older affected individuals suggest later photoreceptor damage. However, the ERGs were normal in these individuals, suggesting a lack of widespread photoreceptor dysfunction despite the fundus and FAF appearance. This combination of clinical, structural, and functional changes does not fit any previously described genetically determined HMD phenotype. Also, family members who were diagnosed with HMD as young adults were asymptomatic. This suggests that though the disease begins at an early age, vision loss occurs only when the fovea is involved, as seen in older affected individuals.

### Photoreceptor rosette formations and RPE damage in retinal degeneration.

At P28, mouse retinal regions electroporated with the chimeric or native *ALOX15* transcripts demonstrated photoreceptor rosettes, which disappeared by P56, resulting in ONL thinning. Multiple mouse models of retinal degeneration display photoreceptor rosettes between P8 and P28 (*Nr2e3*^rd7/rd7^, *Crb1*^–/–^, and *Nrl*^−/−^ mice) and subsequent ONL thinning (55–58). Hence, the rosettes seen in our experiments indicate early photoreceptor degeneration. Further, these changes were absent when a near-null variant (Thr560Met) transcript of *ALOX15* was electroporated. These results collectively suggest that overexpression of catalytically active *ALOX15* alone is sufficient to cause the observed retinal photoreceptor changes in mice, and a similar mechanism could underlie the human HMD phenotype.

Additionally, RPE electroporation with the chimera construct resulted in cell damage. These findings are consistent with the abnormal morphology and membrane discontinuity observed in the RPE of retinal degeneration mouse models (rd9 [*Rpgr*^rd9^], rd10 [*Pde6b*^rd10/rd10^], *Rho*^tvrm4/+^, *Pde6g*^–/–^) (59–61).

The OCT images from 1 patient showed rosette-like circular formations as seen in the electroporated mice. Rosettes have previously been described in other human IRDs (62–64). Further, the ONL thinning seen in older affected individuals resembled the findings in mice at P56, supporting the value of our model. Thinning of macular ONL is also observed in other HMDs (*ABCA4*- and *ELOVL4*) (1). RPE involvement was evident in the OCTs of young (deposition) and older affected individuals (thinning), as well as in electroporated mouse retinas. Taken together, we hypothesize that in human retina, ectopic overexpression of *ALOX15* results in damage of RPE followed by that of the photoreceptors. Further studies are required to determine if photoreceptor damage is secondary to severe RPE damage, *ALOX15* overexpression in photoreceptors, or a combination thereof.

### Chimeras implicated in human diseases.

To our knowledge, this is the first report of a fusion gene underlying IRD. Fusion genes are primarily known to be associated with cancer, and *ZZEF1* has been found to create fusions with other genes in various cancers (65, 66). Rarely, fusion genes have been reported in patients with intellectual disabilities, brain malformation/ocular coloboma, and schizophrenia (67–69).

In the family studied, the chimeric RNA transcript was generated by tandem duplication. This generated a breakpoint in *ZZEF1* intron 6, creating a splice donor site for which the main splice acceptor was the shorter *ALOX15* transcript isoform (NM_001140.3), resulting in a fusion gene. Intriguingly, 12 transcripts were identified, and 2 of the most abundant isoforms (56.8% of the transcripts) were in frame and are plausible candidates to cause the phenotype. Our study adds to the body of literature identifying fusions to be disease-causing entities in noncancerous conditions.

### Neutral lipid alterations related to HMD.

Studies on fatty liver disease in *Alox15*-knockout mice showed decreased liver fat (25), highlighting the possibility of neutral lipid derangements in our HMD phenotype. Patients’ LCLs have higher levels of lipid droplets compared with unaffected family members. While the retina was not directly assessed, we believe this to be an indirect effect of increased ALOX15 activity and a contributor to the HMD subtype. *ELOVL4* and *ABCA4* are previously described HMD subtypes involving lipid dysregulation (70).

### Conclusion.

We identified a chr 17 tandem duplication in a family with a rare, slow, progressive HMD phenotype. This duplication was found to generate a *ZZEF1*-*ALOX15* fusion gene that upregulates *ALOX15* and was confirmed to be pathogenic by in vivo mouse experiments, causing RPE and photoreceptor damage. The human disease also showed early RPE involvement and late photoreceptor damage. Our work highlights that duplication events can generate fusion transcripts resulting in gene expression well above expected levels for duplication. Further, we showed that the primary component of this transcript, *ALOX15*, is likely ectopically overexpressed in retinal photoreceptors. We believe this to be the first study to associate *ALOX15* with HMD. Since ALOX15 catalyzes multiple PUFAs producing a diverse array of lipid metabolites, an imbalance in the production of lipid mediators with a pro-inflammatory skew could contribute to the underlying retinal damage. In addition, increased consumption of membrane-bound retinal DHA by ALOX15 can also exacerbate photoreceptor disc membrane damage. Further studies are required to verify our hypotheses. To our knowledge, discovery of the chimeric transcript demonstrates a genetic mechanism not yet observed in HMD and IRD. Identification of this chimera transcript required investigation at the RNA level, highlighting that chimera transcripts cannot be detected with genomic data alone, making them more difficult to detect. Fusion genes may contribute to the missing heritability in HMD and IRD in general, and this variant type should be routinely considered when investigating unsolved cases of IRD.

## Methods

### Sex as a biological variable.

Sex was not considered as a biological variable in the pedigree. All available individuals within the pedigree (*n* = 14; 5 males; 9 females) were recruited through the ocular genetics program at HSC after obtaining written informed consent. DNA was extracted from blood or saliva samples in all participants. Six family members (3 affected and 3 unaffected) also consented to generation of LCLs and other downstream research procedures.

### Clinical phenotyping.

Ocular assessments including VA measurements and retinal evaluation were performed in all except 2 unaffected individuals (II-3 and II-5). Three affected individuals (III-5, II-2, II-4) underwent phenotyping that included fundus photography, FAF imaging, OCT, ERG and pattern ERG, and EOG and had follow-up examinations.

### Linkage analysis.

Genotyping was performed on 14 family members using Illumina HumanCore Exome-24 or Illumina InfiniumOmni2-5 Exome chip. Subsequently, 14,141 SNPs with unique map positions that had a minor allele frequency > 0.4 (1000 Genomes Project European super-population) and low pairwise linkage disequilibrium (*r*^2^ < 0.05) were used to perform multipoint linkage analysis (Supplemental Methods ii). The maximum lod score was observed under an AD model assuming 99% penetrance, 0.001 allele frequency, and a phenocopy rate of 0.2%.

### GS and analysis.

The DNA extracted from 2 affected members (II-2 and III-5) were genome sequenced using Illumina HiSeq X platform at The Center for Applied Genomics, Toronto, Ontario, Canada (Supplemental Methods iii). Transposable elements, SVs, SNVs, and small indels were called and annotated (71). Candidate variant analysis was done assuming AD mode of inheritance. Rare variants (frequency of SNVs and indels ≤ 0.01%, SVs ≤ 1% based on population databases) that were shared between the 2 affected individuals within the linkage region were prioritized. Details of GS, analysis, annotation, and variant prioritization are summarized in Supplemental Methods iii and iv and Supplemental Figure 3.

### Validation and segregation of tandem duplication.

Breakpoints, disjunct mate reads, split reads, and coverage associated with the duplicated region were verified in Integrative Genomics Viewer v2.4.10 (72) (Supplemental Methods v). Reads from the duplication and flanking region (about 300 base pairs upstream and downstream of the duplicated region) were exported and mapped against a predicted model of the duplication built in Geneious Prime 2020.1.1 (73, 74). Segregation was confirmed by Sanger sequencing of the junction of the tandem duplication (Supplemental Table 5).

### Transcriptome and DGE analysis.

RNA from LCLs [3 affected (II-2, II-4, and III-4) and 3 unaffected family members (II-6, III-2, and III-4)] was isolated using RNeasy Plus mini kit (QIAGEN) as per manufacturer’s protocol. Stranded, polyadenylated mRNA libraries were generated and pair-ended sequenced in an Illumina HiSeq 2500 system. RNA extraction and sequencing are detailed in Supplemental Methods vi and vii.

The read count per gene was calculated with featureCounts (subread1.5.3) (75). Read normalization and DGE analysis (genome wide) were done with DESeq2 (76) on RStudio version 3.2.3 (77). Genes with both a log_2_fold-change greater than 1 and a Benjamini-Hochberg *P* < 0.05 were defined to be differentially expressed.

### Characterization of chimeric transcripts.

A reference model for a chimeric sequence containing *ZZEF1* (NM_015113.4) exons 1 to 6, followed by the entire *ALOX15* (NM_001140.3) coding sequence, was built in silico on Geneious Prime 2020.1.1 (73) (Supplemental Methods viii). RNA-Seq reads of III-5 were paired and mapped to the reference model using Geneious Prime 2020.1.1.

Patient LCL-derived RNA was reverse-transcribed with random primers with SuperScript IV (Thermo Fisher Scientific) according to manufacturer’s instructions. The integrity and sequence variation of the chimeric transcripts were validated by reverse transcriptase PCR with primers at the *ZZEF1* promoter and *ALOX15* 3′-UTR (Supplemental Methods ix). Characterization of the chimeric transcript variants was addressed by cloning the PCR product of III-5 followed by Sanger sequencing of the inserts of 44 colonies (Supplemental Methods x and Supplemental Table 5).

### ddPCR.

ddPCR was performed using cDNA (Supplemental Methods xii) to compare the expression levels of the *ALOX15*, *ZZEF1*, and chimeric transcripts, between 3 affected and unaffected individuals. Custom primers and probes were designed (Supplemental Table 5) for the *ALOX15* and the chimeric transcript assays (Thermo Fisher Scientific), whereas a commercial assay was used to assess the expression of *ZZEF1* (Hs00932991_m1, Thermo Fisher Scientific). Digital PCR was performed on the QX200 ddPCR system (Bio-Rad Laboratories, Inc.) using 40 ng of cDNA. Data were analyzed using QuantaSoft v1.4 (Bio-Rad Laboratories, Inc). Box plots comparing the assays were created based on normalized values (target gene expression/housekeeping gene [TATA box binding protein] expression ratio).

### LC-MS and enzyme kinetics.

LC-MS was performed on LCLs (~20 million cells in triplicates) to compare the levels of 12/15-LOX substrates and metabolites in patients (*n* = 3) and unaffected family members (*n* = 3) (Supplemental Methods xv). Data were quantified as previously described (78).

To assess if levels of substrates could be recovered, LCLs (~18 million cells in triplicates) from 2 affected and 3 unaffected family members were incubated with varying concentrations (0 μM and 5 μM) of PD146176 [(6, 11-dihydro-5-thia-11-aza-benzo[a]-fluorene), Cayman Chemical], a selective inhibitor of 12/15-LOX (79), for 6 hours. Cells were pelleted, then washed in PBS, and LC-MS analysis was repeated.

To assess the kinetic properties of the chimeric ALOX15 enzyme, we grew affected (II-4) and unaffected (II-6) patient-derived LCLs (~20 million cells in triplicates) and treated cells with varying concentrations of substrate, AA (0, 10, 20, 30, 40, 60, 80 μM; Cayman Chemical No. 90010) for 1 hour at 37°C. After treatment, cells were pelleted, lipids were extracted, and LC-MS was performed. We plotted the results as a graph of the rate of 15-HETE product formation and velocity against concentration of substrate.

### ORO staining.

Lymphoblast cells (~200,000 cells in triplicates) from 3 affected and 3 unaffected family members were stained using an ORO kit (MilliporeSigma MAK194). Slides were mounted and imaged using Leica DM 2000 microscope, and ORO stain was quantified using ImageJ software (NIH). Both intracellular and extracellular lipid droplets were quantified.

### Mice research.

WT C57BL/6J mice were ordered from The Jackson Laboratory (stock 000664). The entire litter underwent photoreceptor or RPE electroporation at P0 or P1. All eyes that had successful electroporation were imaged and analyzed further.

### Electroporation of mice retina and immunohistochemistry.

Four plasmids, pT2K-CAGGS-IRES-eGFP-*ZZEF1*-*ALOX15* (chimera), pT2K-CAGGS-IRES-eGFP-*ALOX15* (native *ALOX15*), pT2K-CAGGS-IRES-eGFP-*ALOX15-*Thr560Met (*ALOX15*: c.1679 C>T; p.Thr560Met; retains <5% enzyme activity) (80), and pT2K-CAGGS-IRES-eGFP, were prepared for electroporation. The photoreceptor electroporation was performed as previously described (81). At P28, 3 to 4 mice from each group were euthanized, and 1 to 3 mice were euthanized at P56. Additionally, electroporation targeting the RPE was performed using the chimera and GFP plasmids as previously described (82), and 3 to 5 mice were sacrificed at P28. See Supplemental Methods xvi for details on plasmid cloning, electroporation, tissue staining, and imaging.

### 4C.

The LCLs (10 million cells) derived from 2 patients (II-2 and III-5) and 1 unaffected family member (III-2) were prepared in technical duplicates. UMI-4C was performed following previous reports (35) with modifications. The complete protocol (including viewpoint sequences) is detailed (Supplemental Methods xvii and Supplemental Tables 5 and 11). Libraries were sequenced on a NovaSeq 6000 using 150 bp paired-end reads. All UMI-4C processing steps were completed with UMI4Cats R Package using default parameters (83).

### Statistics.

Box plots, volcano plot, and Manhattan plot were created using RStudio v3.6.3. Statistical significance (*P* < 0.05) between comparison groups was calculated on GraphPad Prism v9.2.0, using unpaired 2-tailed *t* test with Bonferroni’s correction as required. LC-MS data are presented as mean ± SD. Statistical comparison of ORO-positive areas was done on GraphPad software 9.2.0, using the Mann-Whitney test (2-sided). Differential contact frequency on UMI-4C was assessed using normalized counts at the level of individual restriction enzyme fragments using Fisher’s exact test (*P* < 0.05).

### Study approval.

The human research was approved by the Research Ethics Board at the HSC, Toronto, Canada, and met the Tenets of the Declaration of Helsinki. Written informed consent was obtained. Mouse research was approved by the Animal Care Committee at the University Health Network, Toronto, Canada. Mice were handled following the mandates set by Ontario’s Animals for Research Act.

### Data availability.

Most raw data are represented in the main text, supplement, and Supporting Data Values XLS file. As consent to publish data online was not received, additional patient data, including deidentified genome sequencing, will be made available upon request to the corresponding author.

## Author contributions

RA and RH designed and performed the majority of the experiments and analyses (genome, RNA-Seq, Western blot, LC-MS) and wrote the manuscript draft. RA started work on the project first and, hence, is named first among joint authors. ET, KA, EH, and AV supervised experimentation and data analysis. AV and EH performed interpretation of clinical features. MDS performed genome analysis. JC, XC, RA, and RH performed mouse electroporation and staining, supervised by PPM. ML, SM, and MDW performed 4C analysis. AT performed electrophysiology analysis. TAP performed ddPCR. NMR performed linkage analysis. EI and CE performed plasmid experimentation design and analysis, respectively. NES contributed to LC-MS analysis. EC contributed to molecular biology techniques. RVSR performed enzyme kinetics analysis. JTM performed ORO analysis and protein modeling. ADP contributed to the statistical analysis and RNA-Seq analysis. AV supervised the overall project and contributed to the manuscript draft. All authors read and made critical edits to the manuscript draft.

## Supplementary Material

Supplemental data

Unedited blot and gel images

Supporting data values

## Version 1 10/22/2024

In-Press Preview

## Version 2 12/06/2024

Electronic publication
