# Supplementary material for: Autosomal-dominant macular dystrophy linked to a chromosome 17 tandem duplication
Source: JCI Insight. 2024 Dec 6;9(23):e178768. doi: 10.1172/jci.insight.178768 (PMC11623951; doi:10.1172/jci.insight.178768)
Supplement: Supplemental data [file jciinsight-9-178768-s093.pdf]

Supplemental Methods

i. Preliminary Genetic Testing

Next Generation Sequencing (NGS) panel-based gene testing was done in II-2 (26 genes, cone-rod dystrophy panel, Prevention Genetics) and II-4 (244 genes, retinal dystrophy v.8 chip test, Casey Eye Institute Molecular Diagnostic Laboratory). Case II-4 also underwent comparative genomic hybridization array Eye gene array v2 (22 AD retinal dystrophy genes, Oxford Gene Technology). Supplemental Table 2 enlists all genes tested in the family.

ii. SNP Genotyping and Linkage Analysis

Genotyping was performed for all 14 family members by The Center for Applied Genomics (TCAG) at HSC, using Illumina HumanCore Exome-24 (547,644 SNPs (Single Nucleotide Polymorphisms); 12 individuals) or Illumina InfiniumOmni2-5 Exome (2, 612, 357 SNPs; 2 individuals). Genotype calls were made by GenomeStudio v2.0 (Illumina). The SNP call rate was determined by PLINK v1.90b5.2 (1) and SNPs with a call rate of less than 100% were removed. The genotype sets from the HumanCore Exome-24 (1) and Illumnia InfiniumOmni2-5 Exome were merged using PLINK v1.90b5.2, keeping shared SNPs. KING was used to confirm familial relationships and FSuite v1.0.3 (2) was used to check for consanguinity. SNPs with Mendelian errors were filtered out using PEDSTATS 0.6.10 (3).

14,141 SNPs with unique map positions that had a minor allele frequency (MAF; 1000 genomes European super-population) > 0.4 and a low pairwise linkage disequilibrium (LD; $r^2<0.05$) were used to perform multipoint linkage analysis. Prior to the analysis, monomorphic SNPs and SNPs with strand ambiguity were filtered out, and only SNPs on autosomes or chromosome X were kept. Five hundred pedigree simulations were performed with SLINK 3.0.2 (4) to select the best model, and the maximum simulated logarithm of odds (LOD) score was reported by Merlin 1.1.2 (5). The observed LOD score was set under the following conditions: 99% penetrance, 0.001 allele frequency, and phenocopy rate of 0.2%, under a dominant mode of inheritance. A larger SNP (171, 334 SNPs) data set, obtained by merging the initial genotyped SNPs along with SNPs from the 1000 Genomes (1000g) (6) project phase 3 data (European population), was used to narrow the boundaries of the linkage regions.

iii. Genome Sequencing (GS), Variant Calling and Annotation

DNA was extracted (~100ng) from whole blood of two affected members (II-2 and III-5). GS was performed at The Center for Applied Genomics (TCAG) as previously reported (7). Libraries were generated with Illumina TruSeq Nano DNA Library Prep Kit. The DNA was fragmented and posteriorly ligated with TruSeq Illumina adapters. Following PCR amplification, these DNA fragments were paired-end sequenced using Illumina HiSeq X, resulting in pair reads of 150 base pairs. The average read depth was about ~40. GS processing was done at The Center for Applied Genomics. Bcl2fastq2 v2.17 (https://support.illumina.com /downloads/bcl2fastq-conversion-software-v2-20.html) was used to do base calling and FASTQC v0.11.4 (8) was used to verify the quality of the data. Reads were mapped to the hg19 genome using HiSeq Analysis Software X (HAS X, Illumina, v2.5.55.1311). Structural variant (SV) calling was done in Canvas v1.1.0.5 (9), Manta v0.23.1 (10), CNVnator v0.3.2 (11), and ERDS (Estimation by Read Depth with SNV (Single Nucleotide Variant)) v1.1 (12). Mobster v0.2.4.1 (13) was used for transposable element insertions (TEs). Single Nucleotide Variant (SNV) and small indels calling was done in Has X (Illumina). Multiple algorithms were used to call structural variants to avoid missing real variants and strengthen a real variant call. SNV and indels were annotated by TCAG using their pipeline, with elements adapted from ANNOVAR (14).

iv. Genome Analysis Pipeline

Filtering of the GS data assuming autosomal dominant (AD) mode of inheritance, established by pedigree analysis and family history was performed to identify the disease-causing variant. Variants shared between the two affected (III-5 and II-2) that were within the linkage region and were rare (frequency of SNVs and indels $\leq$0.01%, SVs $\leq$ 1%) based on population databases: 1000 Genomes (6), Exome Aggregation Consortium database (ExAC) (15), gnomAD (Genome Aggregation Database, v2.1.1 and v3.1) (16), and MSSNG database (17) were retained, and were prioritized if the gene had retinal relevance. An allele frequency (AF) cut-off of 0.01% was chosen for SNVs and indels, based on the prevalence of the most common HMD subtype (Stargardt's disease) is 1:8,000-1:10,000 (18, 19), and that this family's HMD phenotype is rarer. SNVs with strong functional relevance were kept such as loss of function, frameshift indels, stop-loss, canonical splicing, and epigenetic variants (variants overlapping or near a DNase

hypersensitivity site in human embryonic retina or human RPE cells (20-22); public data complied with Wilson lab, data accession codes: ENCBS941TEO, ENCBS386PSR, ENCBS277FWQ, ENCBS704PVR, ENCBS797BYT). Missense SNVs and intronic variants were kept if they had a significant score in 2 out of 8 predictive algorithms for pathogenicity and conservation: SPIDEX (splicing index score) (23), Polyphen (24), Sorting Intolerant From Tolerant score (SIFT) score (25), Protein Variation Effect Analyzer (PROVEAN) (26), Combined Annotation Dependent Depletion (CADD) Phred score (27), mutation assessor (MA) score (28), PhyloPVert (29) and PhyloPMam (29). Variants with a significant SPIDEX score or synonymous and intronic variants were kept if they had a significant splicing score in SpliceAI (30) or significant scores in one of the splicing predictors compiled by Alamut Visual v2.8.1 (31): SpliceSiteFinder-like (SSF) (32), MaxEntScan (MaxEnt) (33), GeneSplicer (34), Splice Site Prediction by Neural Network (NNPLICE) 0.4 (35), and Human Splicing Finder (HSF) (36). Further details on the GS filtering pipeline are presented in Supplemental Figure 1.

v. Validation and Segregation of the Tandem Duplication

Integrative Genomics Viewer (IGV) v2.4.10 (37) was used to visualize the region of the duplication to confirm the duplication calls made by Canvas v1.1.0.5 (9), Manta v0.23.1 (10), CNVnator v0.3.2 (11), and ERDS (12). Further inspection of the duplication via IGV consisted of assessing: split reads using BLAT (38), discordant read pairs, (pair reads that mapped further apart than expected or in the incorrect orientation) and read coverage to determine the orientation and breakpoints of the duplication. Subsequently, Sanger sequencing was performed to confirm segregation of the tandem duplication. The junction of the duplication was PCR amplified for all 14 family members using primers p5719 and p5720 (Supplemental Table 5) with AllTaq DNA Polymerase (Qiagen). Sanger sequencing of PCR products was performed by TCAG. Geneious Prime 2020.1.1 (39) was used to the align trace files to a custom model of the tandem duplication.

vi. Cell Culture

In a 37ºC incubator at 5% CO_2, Epstein–Barr virus (EBV) transformed lymphoblast (generated by TCAG, Hospital for Sick Children), derived from three affected patients (II-2, II-4, and III-5)

and three unaffected family members (II-6, III-2, and III-4) were grown in RPMI media (Wisent Inc), supplemented with 10% fetal bovine serum (FBS) (Gibco) until confluent (~1x10^6cells/ml).

vii. RNA Extraction and RNA Sequencing

RNA was isolated from lymphoblast samples (~ 15x10^6 lymphoblast cells) of 3 affected family members (II-2, II-4, and III-5) and 3 unaffected family members (II-6, III-2, and III-4) using the RNeasy Plus mini-kit (Qiagen) as per manufacturer's protocol. RNA concentration and quality was assessed on the Agilent 2100 Bioanalyzer system at TCAG, each sample having an RNA integrity number of 10. Stranded, polyadenylated (Poly-A) mRNA libraries were generated at TCAG for each sample with the NEBNext® Ultra™ II Directional RNA Library Prep Kit and NEBNext Poly(A) mRNA Magnetic Isolation Module, using 200 ng of RNA per sample. The cDNA was fragmented into ~300 bases and tagged with Illumina Universal Adapters. The libraries were then sequenced as a multiplex using the Illumina HiSeq 2500 system, generating per sample ~43-51 million, 126 bp paired-end reads. The reads for each sample were assessed for quality using FASTQC 0.11.8 (8). RNA-seq reads were then trimmed using Trimmomatic 0.36 (40) to clip the adaptors and areas of poor quality, using the settings: "ILLUMINACLIP: TruSeq3-PE-2.fa:2:30:10:5:true, LEADING:5, TRAILING:5, SLIDINGWINDOW:5:21, and MINLEN:25". Alignment of the trimmed RNA-seq reads to the hg19 reference genome (41) was done with STAR 2.6.0c (42), sorting the Binary Alignment Map (BAM) by coordinates with default parameters, using two-pass mapping mode. Samtools 1.6 (43) was used to generate the BAM index files.

viii. In Silico Prediction of Chimeric Transcripts

The RNA seq reads of *ZZEF1*, a gene at one of the breakpoints of the duplication and of *ALOX15*, a duplication gene nearest to other duplication breakpoint were inspected on IGV v2.4.10 (37). The BLAT tool (38), implemented on University of California, Santa Cruz (UCSC) portal, was used to identify the location of the observed split reads for *ZZEF1* and *ALOX15* corresponding to the hg19 genome. To construct a reference model, a chimeric sequence between *ZZEF1* and *ALOX15* was built *in silico* on Geneious Prime 2020.1.1 (39), containing *ZZEF1* exon 1 to 6, followed by the entire *ALOX15* (NM_001140.3) coding sequence (CDS) starting at exon 1. Geneious was also used to predict the size of the chimeric transcript

and predict if the transcript was in frame by translating the CDS (coding sequence). RNA-seq reads of one affected patient (III-5) were paired and mapped to the built chimera reference using the RNA-seq mapping option on Geneious Prime 2020.1.1 (39), under default settings to validate the reference.

ix. Reverse Transcription of RNA

The patient-derived lymphoblast RNA was reverse transcribed to cDNA, initially setting up a 20ul reactions containing: 1.2 µg to 2 µg of RNA, 1 µl of 10 mM dNTP (Thermo Fisher Scientific 18427-013), and 1 µl of 100 ng/µl random primers (Thermo Fisher Scientific 48190-011) that were heated at 65°C for 5 min and immediately chilled for 1 minute. Subsequently, 1x SuperScript IV (SSIV) First Strand buffer (Thermo Fisher Scientific 18090010), 5 mM of dithiothreitol (DTT; Thermo Fisher Scientific 18090010), 1 µl of RNaseOUT Recombinant Ribonuclease Inhibitor (40 unit/µl) (Thermo Fisher Scientific 10777-019), and 200 units of SSIV Reverse transcriptase (Thermo Fisher Scientific 18090010) were combined with the primed RNA, and thermo cycled under the following conditions: 23°C for 10 min, 50°C for 30 min, 80°C for 15 min, and at 4°C for 5 min.

x. Segregation of the Chimeric Transcript and Analysis of Alternative Transcript Isoforms

Based on the template of the *ZZEF1-ALOX15* chimeric transcript (chimera) built *in silico*, a primer pair (p5516 and p5578; Supplemental Table 5) was designed to amplify the chimeric transcript from before the start codon of *ZZEF1* till after the termination codon of *ALOX15*. 60.28 ng -100 ng of cDNA from the 3 affected (III-5, II-2, II-4) and 3 unaffected (III-2, III-4, II-6), diluted in a 1:2-1:6 ratio with water was combined with a 12.5 µl master mix containing: 1X SuperFi Buffer (Thermo Fisher Scientific, 12351010), 0.2mM dNTP mix, 0.5µM of each primer, 1X SuperFi GC Enhancer, 0.25 units of Platinum SuperFi DNA polymerase, and water. Cycle conditions were: 98°C for 30 sec, followed by touchdown of 5 cycles 98°C for 10 sec, 65°C - 60°C for 10 sec (1°C decrease in temperature every cycle), and 72°C for 1:50 min, 35 cycles of: 98°C for 10 sec, 60°C for 10 sec, 72°C 1:50 minutes, and a final extension at 72°C for 5 min. Reactions were loaded on 0.8% agarose gel to isolate the PCR products, running at 110 volts for ~50 min in tris acetate (TA) 1x buffer. Bands of interest (chimera, ~3500 bp) were excised and

cDNA isolated by centrifugation (10,000 RPM for 10 min). Sanger sequencing was conducted at TCAG. Trace files were aligned to chimera reference on Geneious Prime 2020.1.1 (39).

To generate the cloning insert, 100 ng of cDNA from affected individual, III-5 (diluted in a ~1:5 ratio with water) was PCR amplified in ten, 12.5ul reactions, using the same primers, cycle conditions, and electrophoresis protocol. Bands matching the chimera size were recovered from the gel, and the DNA was purified using the QIAEX II gel extraction kit (Qiagen) following the manufacturer's instructions. 408 ng of the purified PCR product was ligated to a pJET1.2 vector using the CloneJET PCR cloning kit (Thermo Fisher Scientific). 140 ng of the ligated vector was transformed into One Shot Stbl3 cells (Thermo Fisher Scientific) as per manufacturer's guidelines. In total, 44 colonies were grown, then purified with the QIAprep Spin Miniprep kit (Qiagen), and Sanger sequenced to characterize the chimeric transcripts and their corresponding frequencies. The full length of the inserts was Sanger sequenced at TCAG with the following primers: pJET forward, and pJET reverse (Thermo Fisher Scientific), p5516, p5518, p5578, p5581, p5582, p5670, p5772, p5773, and p5673 (Supplemental Table 5).

xi. Allele-Specific Expression

Using IGV (37) v2.4.10, we searched the GS data of patient II-2 and III-5 for heterozygous exonic SNPs, that were present in either *ZZEF1* exons 1-6 or *ALOX15*. Subsequently, SNPs that met these criteria were assessed on IGV and compared to the RNA-seq data to assess if expression deviated from the genomic data. Subsequently, PCR and Sanger sequencing was performed to validate the SNPs in all 14 family members.

xii. Droplet Digital PCR

Droplet digital PCR (ddPCR) was performed to compare the expression levels of the *ALOX15*, *ZZEF1*, and chimeric transcripts. Custom primers and probes (Thermo Fisher Scientific, 4400294) were designed at TCAG for the *ALOX15* and the chimeric transcript assays. To detect the chimeric transcript, primer and probe positions were designed to account for most of the isoform variations detected by cloning. Thus, a probe and forward (FWD) primer were designed on *ZZEF1* exon 5, and a reverse (RVS) primer designed on *ALOX15* exon 1. To quantify gene expression of the *ALOX15* transcripts (NM_001140.3 and ENST00000570836.1), primers were

designed spanning the region from the exon 3-4 boundary to near the end of exon 4, with a probe on exon 4. To assess the expression of *ZZEF1*, a commercial assay (Hs00932991_m1, Thermo Fisher Scientific) was used, spanning the region between exon 29 and 30 with the probe in the boundary. A Chimera 1 plasmid (the most common isoform) was used as positive control for the *ALOX15* and chimera ddPCR assays and as a negative control for the *ZZEF1* assay. Human universal cDNA (Human XpressRef Universal Total RNA, Qiagen, Hilden, Germany, 338112) expresses ZZEF1 and ALOX15 well, thus served as a positive control for both. *TBP* (TATA-box binding protein) (Hs00427620_m1 assay, Thermo Fisher Scientific) was used as a housekeeping gene.

xiii. Western Blot

Western blot was performed to ascertain if the chimeric transcript was expressed at the protein level. The lymphoblast cells (~15 million) were lysed in RIPA buffer, and protein lysates (~30ug per sample) were loaded on a 7% Nupage Tris-Acetate Gel and transferred onto a PVDF membrane. After transfer, membrane was blocked in 5% milk in TBS/0.1% Tween (TBST) and subsequently incubated with the primary (Alox15: Abcam ab244205 1:1000, β-actin antibody (Sigma-Aldrich A1978; 1:20,000) and secondary (anti-rabbit HRP, Abcam ab6721 1:7000) antibodies diluted in 3% BSA/TBST. The membrane was imaged on the BioRad ChemiDoc system using the ECL substrate (Cytiva, RPN2232).

xiv. Immunohistochemistry on Human Retina

A fresh human donor eye (49-year-old female with no pre-existing eye conditions) was obtained from the Eye Bank of Canada. Cryosections (16 µm) were placed on Superfrost Plus Microscope Slides (Fisher scientific), washed with PBS (phosphate buffered saline), and then permeabilized by incubation in 0.1% tween in PBS (PBST) for 10 minutes. Slides were blocked with 5% donkey serum (Sigma-Aldrich, St. Louis, MO, USA) in PBST and then incubated with primary antibodies (anti-ZZEF1 HPA031778, Sigma, 1:200, and anti-ALOX15 ab11974, Abcam, 1:100) in 5% donkey serum + PBST, overnight). Secondary antibodies (Alexa Fluor 488 A-21202, and Alexa Fluor 647 A-31573, Thermo Fisher Scientific) were diluted 1:1000 in 5% donkey serum + PBST + DAPI (Sigma D9564) and incubated for 1hr at room temperature. Slides were mounted

with MOWIO and imaged using a 60x oil immersion objective on a Zeiss LSM 880 laser scanner confocal microscope.

xv. Liquid Chromatography Mass Spectrometry (LC-MS)

Cultured lymphoblast cells (~20M) from 3 unaffected and 3 affected family members were washed twice with cold Phosphate Buffered Saline (PBS) and pelleted. Cell pellets were resuspended in 1 mL ultra-pure water and sonicated for 1 minute on ice using Fisher Scientific Sonic Dismembrator 550. Pellets were then spiked with a mixture of deuterated analogs (Arachidonic Acid-d_8 and 15(S)-HETE-d_8, Cayman Chemical, MI, USA) of lipid mediators (1 ng each), acidified to pH 3 with 0.1 N HCl and extracted three times with 4 mL ethyl acetate. The ethyl acetate layers were washed to neutrality with water, pooled into a set of siliconized conical tubes and evaporated to dryness under a stream of nitrogen. Residues were reconstituted into 100 µL acetonitrile/water/acetic acid (30/70/0.02) and transferred to siliconized micro-inserts for analysis. Lipid mediators were measured by LC-MS using a QTRAP 5500 triple-quadruple mass spectrometer (Sciex: Framingham, Massachusetts, USA) in negative electrospray ionization mode by MRM data acquisition with an Agilent 1200 HPLC (Agilent Technologies: Santa Clara, California, USA). Chromatography is performed on a Kinetex C18 column (100 x 4.6 mm, 5 µ particle sizes) (Phenomenex, California). The HPLC flow is maintained at 800 µL/minute with a gradient consisting of: A= Water/Acetonitrile (90/10) + 0.02% acetic acid and B = Acetonitrile/Isopropanol with a total run time of 19 minutes.

Standard curves of lipid mediators were generated from calibration mixes from 0.01 ng to 10 ng. Data was quantified by plotting the sample peak area ratios of individual lipid mediators against their corresponding standard curve in Multiquant 3.0.1 software (ABSciex : Framingham, Massachusetts, USA).

xvi. Electroporation of the Mice Retina

Plasmid Cloning: The most dominant *ZZEF1-ALOX15* chimera isoform cloned by the pJET vector (Chimera 1) was PCR amplified, adding *Xho*I and *Not*I overhangs (primers p5685 and p5686, Supplemental Table 5). The purified PCR product was cloned into the pJET 1.2 vector (Thermo Fisher Scientific), followed by extraction of the plasmid DNA using the QIAprep spin

miniprep kit (Qiagen). The plasmid was digested by *Xho*I and *Not*I enzymes and cloned into a pT2K-CAGGS-IRES-eGFP vector (44, 45) that had also been restriction digested by XhoI and NotI, generating a pT2K-CAGGS-IRES-eGFP-*ZZEF1-ALOX15* plasmid. This plasmid subsequently underwent site-directed mutagenesis to generate an ALOX15 plasmid with the missense variant NM_001140.5: c.1679 C>T; p.Thr560Met (rs34210653). 10 ng of the pT2K-CAGGS-IRES-eGFP-ALOX15 plasmid were amplified in a 10 uL reaction with 10 uM of primers specific to the missense variant (Thr560Met F, Thr560Met R, Supplemental Table 5) and the KAPA HiFi DNA Polymerase (Roche) for 18 cycles at an annealing temperature of 68℃. After amplification, three reactions were pooled together and treated with DpnI for 1 hour at 37℃. 10 uL of the DpnI-treated product was transformed into Stbl3 competent cells. To isolate the mutant plasmid DNA, the QIAprep spin miniprep kit (Qiagen) was used. The isolated plasmid was cloned into a pT2K-CAGGS-IRES-eGFP vector as described above, generating a pT2K-CAGGS-IRES-eGFP-ALOX15-Thr560Met plasmid. Lastly, four plasmids, pT2K-CAGGS-IRES-eGFP-*ZZEF1-ALOX15*, pT2K-CAGGS-IRES-eGFP-*ALOX15*, pT2K-CAGGS-IRES-eGFP-ALOX15-Thr560Met, and pT2K-CAGGS-IRES-eGFP (empty control plasmid), were purified with the Endofree plasmid Maxi kit (Qiagen). All plasmid sequences were validated by Sanger sequencing at TCAG. Plasmid expression was confirmed in HEK293T (CRL-3216, ATCC) cells.

Injections: The electroporation experiment was based on protocols outlined in prior publications (46-49). Ice was used to anesthetize mice for the procedures. Electroporation was performed on the right eye of P0 or P1 mice as follows: observing under a surgical microscope, a 30 -gauge needle was used to make an incision in the sclera at the sclerocorneal junction. Then, using a 10 µl Hamilton syringe with a blunt end 0.5″ 32-gauge needle tip, a 0.3ul subretinal injection of DNA solution was delivered. This solution contained 0.1% fast green dye to help guide the needle into the correct location. 10 mM sized tweezer electrodes were then used to apply 5 electrical pulses at 80 V for 50 ms with 950 ms intervals between pulses, to facilitate DNA entry into the cells. Electroporation was directed toward the mitotic retinal progenitor cells, thus the majority of cells generated from these progenitors are rods (46). The following constructs were used to generate four mice groups: pT2K-CAGGS-IRES-eGFP-*ZZEF1*/*ALOX15* (Chimera 1 plasmid), pT2K-CAGGS-IRES-eGFP-*ALOX15* (native *ALOX15* plasmid), pT2K-CAGGS-IRES-eGFP-ALOX15-Thr560Met (near null *ALOX15* plasmid), and pT2K-CAGGS-IRES-eGFP

(control plasmid). After the procedure mice were placed beneath a heating lamp to recover from the anesthesia.

To determine if chimera expression is damaging to RPE cells, a similar injection protocol was followed to electroporate the RPE, but instead we applied two pulse trains consisting of 5 electrical pulses at 80 V for 5 ms with a 1 s interval between pulses with the positive electrode positioned on the non-electroporated eye (50). For the RPE electroporation, only the Chimera 1 and control plasmids were used.

Tissue preparation: Following photoreceptor electroporation, mice were euthanized at P28 (n=3-4) and P56 (n=1-3). Eyes were fixed in 4% paraformaldehyde in PBS. Under a dissecting microscope an incision was made in the cornea and the lens was removed. Eyes were then placed in 30% sucrose in PBS overnight at 4°C. Eyes were then put in a solution of 50% OCT media (Sakura Finetek, Torrance, CA, USA) and 15% sucrose in PBS for 30 min. Blocks were placed in OCT media and were frozen over dry ice and stored at -80°C.

Following RPE-electroporation, chimera (n=5) and GFP-electroporated (n=3) mice were euthanized at P28 and an RPE flat mount was prepared as previously described (51). Following overnight fixation, the retina, iris, ciliary body, and lens were removed, leaving only the RPE, cornea and sclera. Micro scissors were used to make four radial cuts to flatten the eye, and the RPE was subsequently stored in PBS at 4˚C protected from light until ready for staining.

Immunohistochemistry: For P28 and P56 mice, 14μm thick cryosections were placed on Superfrost Plus Microscope Slides (Fisher Scientific). Slides were washed in PBS and blocked in 10% FBS in PBST for 1hr and then incubated overnight at 4C with primary antibodies for Cone Arrestin (Millipore-Sigma; AB15282; 1:1000), Rhodopsin (Sigma-Aldrich; O4886, 1:10,000), GFP (Abcam; ab13970; 1:2000) diluted in blocking solution. The next morning, slides were washed and incubated with secondary antibodies (Alexa Fluor 488 A78948, Alexa Fluor 555 A-31570, and Alexa Fluor 647 A-31573, Thermo Fisher Scientific) diluted 1:1000 in 5% donkey serum + DAPI (Sigma D9564) for 1hr at room temperature. After mounting (MOWIOL media), slides were imaged at 20x on the Zeiss LSM 880 confocal microscope using the Zen (Zeiss) software.

For the RPE flat mount, the tissue was washed twice in 0.5% bovine serum albumin (BSA) and 0.1% Triton in PBS, then blocked in 0.5% BSA + 5% DS + 0.1% triton in PBS for 20 minutes at RT. The RPE was then incubated for 1 hour at room temperature with primary antibodies for RPE65 (Abcam; ab231782; 1:250) and GFP (Abcam; ab13970; 1:2000) diluted in blocking serum. The tissue was washed and incubated with secondary antibodies (Alexa Fluor 488 A78948 and Alexa Fluor 555 A-31572, Thermo Fisher Scientific) diluted 1:500 in 5% donkey serum + DAPI (Sigma D9564) for 1hr at room temperature. Following washes, RPE was incubated for 15 minutes with Phalloidin-iFluor 647 (Abcam; ab176759; 1:400). RPE was then washed and mounted onto slides for imaging. All slides were imaged at 20x on the Zeiss LSM 880 confocal microscope using the Zen (Zeiss) software.

xvii. Chromosome Conformation Capture

Primer Design: Each primer was designed following the guidelines established in Schwartzman et al. (2016) (52). Upstream primers were designed to target 50-100 bp upstream of the downstream primer which resides near a DpnII (GATC) site. For allele specific viewpoints, each downstream primer targeted a region 10-30 base pairs away from a DpnII site, whose pad sequence (sequence between the GATC and the 3’ end of the downstream primer) contained an SNP to allow for allele specific read mapping. Primers were all designed to have a Tm of 58°C with sequences compatible for multiplexing. For the complete list of primers see Supplemental Table 5.

Generation of 3C template: 10 million patient or control derived LCL (Lymphoblast Cell Line) cells were harvested for each biological replicate (2 patient and 1 WT sample). Each group of cells was crosslinked in a 2% formaldehyde solution for 10 min, before being quenched in 0.125 M glycine. Cells were centrifuged (600xg for 5 min at 4°C for each wash step in the generation of 3C template) and washed in 1 mL PBS. Cells were split into two technical replicates per sample. Cells were subsequently lysed in 5mL lysis buffer (50 mM Tris–HCl, pH 7.5, 150 mM NaCl, 5 mM EDTA, 0.5% NP-40, 1% TX-100, supplemented with 1X protease-inhibitor) and incubated on ice for 30 min, with gentle agitation. Nuclei were then washed in 1 mL PBS before being transferred to a new 1.5 mL Eppendorf tube. Nuclei were resuspended in 450 µL of nuclease free H_2O. To permeabilize nuclei, 15 µL 10% sodium dodecyl sulfate and 60 µL

NEB3.1 buffer were pre warmed to 37°C and added to the samples and incubated for 37°C at 900 rpm for 1 hour in a Thermomixer (Eppendorf). Next, 75 µL of prewarmed 20% Triton-X 100 was added and incubated for an additional 1 hour. A 10 µL aliquot was taken from each sample to check for DNA integrity (undigested control). After permeabilization 200 units of DpnII (NEB, R0543M) was added to each sample and they were incubated at 37°C, and 900 rpm for 4 hours. Afterwards, an additional 200 units of DpnII were added, and nuclei were incubated an additional 16 hours. Digestion reactions were stopped via heat-inactivation of DpnII according to manufacturer's protocol. An 8µL aliquot of each sample was taken to check for digestion efficiency (digestion control). Nuclei were then washed 2 times with PBS before being resuspended in 415 µL nuclease-free H_2O (nf H_2O). To ligate the digested nuclei 50 µL of 10x T4 Ligase Buffer, 5 µL of 10 mg/mL BSA, and 10000 units of T4 DNA ligase (NEB, M0202T) were added to the digested nuclei and the samples were adjusted to 500 µL with nf H_2O. Ligations proceeded overnight at 16°C. After ligation, a 10 µL aliquot of each sample was taken to check for ligation efficiency (ligation control). Nuclei were then washed 2 times with PBS and resuspended in 250 µL of 10 mM Tris-HCl, pH 7.5. All control aliquots were adjusted to 100 µL with Tris-HCl, pH 7.5. To reverse crosslink each sample and control was subjected to 1:40 v/v dilution of 10 mg/mL Proteinase K and incubated at 65°C overnight. Proteinase K was heat-inactivated by incubation at 85°C for 20 min. To remove RNA 1:20 v/v dilution of 10 mg/mL RNAse was added to each sample, and they were incubated at 45°C for 45 min. To isolate DNA 0.5X AMPure XP beads were used according to manufacturer's instructions and eluted in 32 µL EB (10mM Tris-HCl, pH 8.5) for the samples, and 10 µL EB for each control. Controls were run on a 0.6% agarose gel to check for a smear in the digested controls, and higher MW bands in the ligation controls.

Creation of UMI-4C library: 3-5 µg of 3C template was diluted in 200 µL of EB and sonicated in a Diagenode Biorupter® Pico using a program of 30s on, 60s off for 5 cycles. End repair (NEB E6050S) was performed according to the manufacturer's protocol. End repaired DNA was cleaned up with 2X AMPure XP beads and eluted in 75 µL nf H_2O. A-tailing (NEB, E6053) was completed according to manufacturer's protocol, and heat inactivated at 75°C for 20 min. Samples were then treated with Quick-CIP (NEB M0525S) according to manufacturer's protocol, and heat inactivated at 80°C for 2 min. Samples were cleaned up using 2X AMPure XP beads and eluted in 60 µL nf H_2O. To add adaptors, 80 µL 2x Quick-ligase buffer (NEB, M2200S), 10

uL Quick-ligase, and 5 µL of custom 15 µM Illumina compatible adapters were assembled and incubated at 25°C for 25 min. Reactions were heat inactivated at 96°C for 5 min. Finally, samples were cleaned up with 0.8X AMPure XP beads and eluted in 32 µL nf H_2O. To quantify samples 1µL aliquots of DNA were diluted 5X and heated to 95°C for 2 min to produce ssDNA. ssDNA was measured using Qubit 2.0 fluorometer according to manufacturer's instructions.

To generate sequence-ready libraries, two rounds of PCR were completed. The first PCR using the universal reverse primer and multiplexed locus specific upstream primers was completed using Kapa HotStart (Roche, 7958927001). 5X 50 µL reactions were assembled for each viewpoint containing 50 ng of library and 0.4 mM forward and reverse primers. Samples were run as follows: 1 cycle of 95°C for 2 min, 18 cycles of 98°C for 20 s, 60°C for 15 s, and 72°C for 1 min, and a final cycle of 72°C for 1 min. Reactions were cleaned with 1.8X AMPure XP beads and eluted in 21µL. The subsequent PCR contained the universal reverse primer and the multiplexed downstream locus specific primers. Samples were run with the same cycling conditions. The 5 reactions for each sample were pooled and cleaned up using a two-tailed AMPure XP beads cleanup (0.5X, then 1.3x) and final libraries were eluted in 21 µL 0.1X TE. Libraries were Quantified using the Quibit™ dsDNA HS Kit (ThermoFisher, Q32851) and analyzed on an Agilent 2100 bioanalyzer to assess insert sizes.

Sequencing, and bioinformatic analysis: Libraries were sequenced on a NovaSeq 6000 S4 flowcell using paired-end 150 bp chemistry. Each library was sequenced to a depth of 50-60 million reads. Analysis of UMI-4C data was performed using the R package UMI4Cats (53) as described below:

Reference genome: A digested hg19 reference was generated following authors' recommendations for DpnII (RE seq=GATC): https://tanaylab.github.io/umi4cpackage/articles/umi4c-newDB.html. A 50bp mappability track was generated using gemtools v1.7.1.

Demultiplexing and resolution of combined/allele-specific viewpoints: Multiplexed fastq files were split into single viewpoints using the demultiplexFastq function from UMI4Cats, using the downstream primer + pad sequence as the 'barcode'. Allele-specific substitutions were included at this stage to obtain allele-specific viewpoint libraries.

Pre-processing, Mapping and UMI counting: Valid bait fragments were identified and split using the prepUMI4C and splitUMI4C functions, which splits multi contact reads into individual fragments based on the RE sequence. Mapping to the digested hg19 genome was performed using the alignmentUMI4C function using default settings. Raw collapsed UMI counts were obtained per viewpoint using the counterUMI4C function. Counts from patient samples were normalized to reflect copy-number for viewpoints within the duplicated region (divided by 2 for disease allele-specific or divided by 1.5 for allele-agnostic viewpoints and rounded up to the nearest integer). Normalized counts were used for subsequent plotting of domainograms and evaluation of differential contact frequencies. Allele-specific domainograms were calculated by normalizing to the wildtype allele, using a quotient-based normalisation method.

Supplemental Table 1. Clinical Phenotype of the affected participants.

	**III-5**	**II-4**	**II-2**	**II-7**	**III-1**
**Current Age/Sex**	31/F	67/F	70/F	60/F	25/F
**Age of Diagnosis**	24	61	25	40s	18
**Onset of Symptoms**	None	Mild photophobia (age 61)	Decrease distance vision (age 56)	Mildly reduced vision	None
**VA (Visual Acuity)**	20/25 and 20/20	20/40 and 20/25	20/800 and 20/40	20/50 and 20/50	20/25 and 20/20
**Colour HRR (Hardy Rand Rittler chart)**	Normal Red Green (RG) & Blue Yellow (BY)	Normal RG & BY	Mild RG and BY deficit	Normal RG & BY	N/A
**Fundus (OU)**	Disc: Normal; Macula: concentric deep retinal hypopigmented RPE changes in the para and perifoveal and paramacular regions	Disc: peripapillary atrophy; Macula and mid-peripheral retina show extensive deep retinal hypo-pigmented RPE changes with some atrophy, dull foveal reflex.	Disc: Normal; Macula and mid-peripheral retina show extensive deep retinal hypopigmented RPE changes with some atrophy; macula is atrophic in the right eye and shows dull foveal reflex in the left eye.	Disc: normal Macula: Deep retinal hypopigmented RPE changes in the macula extending up to the arcades	Diagnosed with HMD at 18 years (Ottawa Hospital)

**ERG, PERG (Pattern ERG) and electro-oculogram (EOG)**	Normal rod and cone ERG responses; Normal PERG; Normal EOG	Normal rod and cone ERG responses; PERG shows peripheral macular dysfunction; Normal EOG	Normal rod and cone ERG responses; PERG shows central and peripheral macular dysfunction; Normal EOG	N/A	N/A
**Visual Fields (OU: both eyes)**	GVF: (Goldmann visual field): normal fields to I4e and III4e targets	GVF: preservation of central 10° fields to I4e target surrounding which there is a scotoma; preservation of 105° fields to III4e target	GVF right eye (OD) central scotoma 60° (vertical) x 60° (Horizontal) to I4e target<br><br>GVF left eye (OS) central scotoma 55° (vertical) x 25° (Horizontal) to I4e target	N/A	N/A

Supplemental Table 2. Genes tested in different clinical genetic tests done for II-2 and II-4

**Case II-2 Cone-Rod Dystrophy Next Generation Sequencing Gene Panel (26 Genes)**						
ABCA4	*ADAM9*	*AIPL1*	*C8orf37*	*C21orf2*	*CABP4*	*CDHR1*
CNNM4	*CRX*	*CACNA1F*	*CACNA2D4*	*GUCA1A*	*GUCY2D*	*KCNV2*
CERKL	*CNGB3*	*PDE6C*	*PITPNM3*	*PROM1*	*PRPH2*	*RDH5*
RAX2	*RIMS1*	*RPGRIP1*	*SEMA4A*	*UNC119*		
**Case II-4 Gene Array v2 Autosomal Dominant Retinal Dystrophy Genes (22 Genes)**						
PRPF3	*PRPF31*	*PRPF8*	*PRPF6*	*PRPH2*	*BEST1*	*CRX*
FSCN2	*GUCA1B*	*IMPDH1*	*KLHL7*	*NR2E3*	*NRL*	*RDH12*
RHO	*ROM1*	*RP1*	*RP9*	*RPE65*	*SEMA4A*	*SNRNP200*
TOPORS						
**Case II-4 Next Generation Sequencing Based Retinal Dystrophy v8 Chip Test Genes (244 Genes)**						
ABCA4	*ABCD1*	*ABHD12*	*ADAM9*	*AHI1*	*AIPL1*	*ALMS1*
AMACR	*ARL2B*	*ARL6*	*ARL13B*	*ATF6*	*B9D1*	*B9D2*
BBIP1	*BBS1*	*BBS2*	*BBS4*	*BBS5*	*BBS7*	*BBS9*
BBS10	*BBS12*	*BCM*	*BEST1*	*C1QTNF5*	*C2orf71*	*C5orf42*
C8orf37	*C21orf2*	*CA4*	*CABP4*	*CACNA1F*	*CACNA2D4*	*CC2D2A*
CDH3	*CDH23*	*CDHR1*	*CEP41*	*CEP164*	*CEP250*	*CEP290/BBS14*
CERKL	*CHM*	*CIB2*	*CISD2*	*CKAP4*	*CLN3*	*CLN5*
CLN6	*CLN8*	*CLN13*	*CLN14*	*CLRN1*	*CNGA1*	*CNGA3*
CNGB1	*CNGB3*	*CNNM4*	*COH1*	*CRB1*	*CRX*	*CSPP1*
CTSD	*CYP4V2*	*DFNB31*	*DGKQ*	*DHDDS*	*DHX38*	*DNAJC5*
DRAM2	*DTHD1*	*EFEMP1*	*ELOVL4*	*EMC1*	*EYS*	*FAM161A*
FSCN2	*FZD4*	*GDF6*	*GJB2*	*GJB6*	*GNAT1*	*GNAT2*
GPR98	*GPR125*	*GPR179*	*GRK1*	*GRM6*	*GRN*	*GUCA1A*
GUCA1B	*GUCY2D*	*HARS*	*HGSNAT*	*HK1*	*HMX1*	*IDH3B*
IFT27	*IFT140*	*IFT172*	*IMPDH1*	*IMPG1*	*IMPG2*	*INPP5E*
IQCB1	*ITM2B*	*KCNJ13*	*KCNV2*	*KIAA1549*	*KIF7*	*KIZ*
KLHL7	*LCA5*	*LRAT*	*LRIT3*	*LRP5*	*LZTFL1*	*LHON*
MAK	*MERTK*	*MFN2*	*MFRP*	*MFSD8*	*MIR204*	*MKKS*
MKS1	*MMACHC*	*MYO7A*	*MVK*	*NDP*	*NEK2*	*NEUROD1*
NMNAT1	*NPHP1*	*NPHP4*	*NR2E3*	*NR2F1*	*NRL*	*NYX*
OAT	*OFD1*	*OPA1*	*OPA3*	*OR2W3*	*OTX2*	*PCDH15*
PDE6A	*PDE6B*	*PDE6C*	*PDE6D*	*PDE6G*	*PDE6H*	*PDZD7*
PEX1	*PEX2*	*PEX3*	*PEX5*	*PEX6*	*PEX7*	*PEX10*
PEX11B	*PEX12*	*PEX13*	*PEX14*	*PEX16*	*PEX19*	*PEX26*
PHYH	*PITPNM3*	*PNPLA6*				
POC1B	*PPT1*	*PRCD*	*PROM1*	*PRPF3*	*PRPF4*	*PRPF6*
PRPF8	*PRPF31*	*PRPS1*	*RAB28*	*RAX2*	*RBP3*	*RBP4*
RD3	*RDH5*	*RDH11*	*RDH12*	*PRPH2*	*RGR*	*RGS9*

RGS9BP	*RHO*	*RIMS1*	*RLBP1*	*ROM1*	*RP1*	*RP1L1*
RP2	*RP9*	*RPE65*	*RPGR*	*RPGRIP1*	*RPGRIP1L*	*RS1*
SAG	*SDCCAG8*	*SEMA4A*	*SLC7A14*	*SLC24A1*	*SNRNP200*	*SPATA7*
TCTN1	*TCTN2*	*TCTN3*	*TEAD1*	*TIMM8A*	*TIMP3*	*TMEM67*
TMEM126A	*TMEM138*	*TMEM216*	*TMEM231*	*TMEM237*	*TOPORS*	*TPP1*
TRIM32	*TRPM1*	*TSPAN12*	*TTC8*	*TTC21B*	*TTLL5*	*TULP1*
UNC119	*USH1C*	*USH1G*	*USH2A*	*WDPCP*	*WDR19*	*WFS1*
ZNF408	*ZNF423*	*ZNF513*				
**Case III-5 Mitochondrial Genes**						
MT-TY	MT-TW	MT-TV	MT-TT	MT-TS2	MT-TS1	MT-TR
MT-TQ	MT-TP	MT-TN	MT-TM	MT-TL2	MT-TL1	MT-TK
MT-TI	MT-TH	MT-TG	MT-TF	MT-TE	MT-TD	MT-TA
MT-RNR2	MT-RNR1	MT-ND6	MT-ND5	MT-ND4L	MT-ND4	MT-ND3
MT-ND2	MT-ND1	MT-CYB	MT-CO3	MT-CO2	MT-CO1	MT-TC
MT-ATP8	MT-ATP6					
**Case III-5 Interactor Nuclear Genes**						
APTX	DGUOK	DNA2	FBXL4	GFER	MGMEL	MPV17
OPA1	OPA3	POLG	POLG2	RRM2B	SLC25A4	SPG7
SUCLA2	SUCLG1	TK2	TWNK	TYMP		

Supplemental Table 3. Boundaries from Genome Wide Linkage Analysis

Chr	Start		End		Length
	SNP	hg19	SNP	hg19	Mb
1	rs10800024	164,457,615	rs6427004	166,399,575	1.9
6	rs10456274	23,469,680	rs2766543	35,708,634	8.6
7	rs6583338	46,239	rs724254	7,186,544	7.1
7	rs1824552	155,693,481	rs7811473	158,947,104	3.3
9	rs6474865	14,869,861	rs224022	20,313,099	5.4
13	rs9560745	91,708,800	rs4148481	95,845,706	4.1
17	rs11867500	2,942,648	rs9915706	6,654,919	3.7
18	rs11875775	73,856,073	rs4799099	77,639,585	3.8
19	rs8104864	13,970,625	rs11086017	16,085,596	2.1
20	rs1078571	39,353,832	rs2299978	43,687,706	4.3
X	rs2227059	138,236,904	rs5925199	151,824,105	13.6

Supplemental Table 4. List of candidate variants remaining after genome filtering.

Variant type	hg19 position: change	Transcript number (if applicable) and Predicted effect	Target genes	Predictive scores or observations
CNV	chr17: 4,012,590-4,573,014 tandem dup	Overexpression of duplicated genes	*ZZEF1*, *CYB5D2*, *ANKFY1*, *UBE2G1*, *SPNS3*, *SPNS2*, *MYBBP1A*, *GGT6*, *SMTNL2*, *ALOX15*	NA
Missense	chr7: 4,899,761:C>T	NM_020144.4: c.1681G>A, p.(Ala561Thr)	*PAPOLB*	• SIFT 0.67 (tolerated), Polyphen 0.005 (benign), PhyloPMam/vert 0.036/0.1 (not well conserved)<br>• No eye phenotype/disease association in the literature<br>• RNA-seq shows non-aberrant gene expression
UTR5’	chr17:5095143: G>C	NM_032530: c.-7592C>G May affect gene expression	*ZNF594*	• PhyloPMam/vert: -0.864/-2.22 (not well conserved)<br>• RNA-seq shows non-aberrant gene expression<br>• No eye disease association in literature
UTR5’	chr6:31093542: G>A	NM_014068: c.-3868G>A May affect gene expression	*PSORS1C1*	• PhyloPMam/vert: -0.364/-0.411 (not well conserved)<br>• RNA-seq shows non-aberrant gene expression<br>• Susceptibility loci (rs12525170; not present in our patients) linked with Behçet's disease [A]
UTR3’	chr6:33740038: C>G	NM_001143944: c.*367G>C	*LEMD2*	• PhyloPMam/vert: 1.068/0.122<br>• RNA-seq shows non-aberrant gene expression<br>• Associated with autosomal recessive juvenile onset cataracts, an eye disease not related to HMD[B]

Intergenic	chr17:5,095,143: G>C	Epigenetic (May affect gene expression)	upstream ZNF594	Distance to DHS: 0 bp, no nearby genes of retinal relevance
Intronic	chr6:35,655,956: G>A	Epigenetic (May affect gene expression)	*FKBP5* intron	Distance to DHS =54 bases, no nearby genes of retinal relevance
Intronic	chr18:74,780,354 TGAC>T	Epigenetic (May affect gene expression)	*MBP* intron	Distance to DHS = 137 bases, no nearby genes of retinal relevance
Intergenic	chr17:5,970,156 C>CTCTA	Epigenetic (May affect gene expression)	N/A	Distance to DHS = 51 bases, no nearby genes of retinal relevance
Intergenic	chr6:28,129,087 T>A	Epigenetic (May affect gene expression)	upstream *ZNF192P1*	Distance to DHS = 362 bases, no nearby genes of retinal relevance

A: Hughes, T., Coit, P., Adler, A. et al. Identification of multiple independent susceptibility loci in the HLA region in Behçet's disease. Nat Genet 45, 319–324 (2013). https://doi.org/10.1038/ng.2551

B: Boone PM, Yuan B, Gu S, Ma Z, Gambin T, Gonzaga-Jauregui C, Jain M, Murdock TJ, White JJ, Jhangiani SN, Walker K, Wang Q, Muzny DM, Gibbs RA, Hejtmancik JF, Lupski JR, Posey JE, Lewis RA. Hutterite-type cataract maps to chromosome 6p21.32-p21.31, co-segregates with a homozygous mutation in LEMD2, and is associated with sudden cardiac death. Mol Genet Genomic Med. 2015 Nov 14;4(1):77-94. doi: 10.1002/mgg3.181. PMID: 26788539; PMCID: PMC4707028

Supplemental Table 5. List of primers and probes organized by experiment.

**Primer ID**	**Sequence 5’ - 3’**
**To segregate chromosome 17 tandem duplication**	
p5719	AAATCTCATGGCTAAAGAGGACTTTTC
p5720	CTGCACAATACTCAGTAAGTCATTCTC
**To validate and segregate chimeric transcripts in 6 family members**	
p5516	CGCCCAAGACGGAGACCC
p5578	TGTGTTCACTGGGTGCAGAG
**To ligate Chimera 1 insert in the pJET vector**	
p5516	CGCCCAAGACGGAGACCC
p5578	TGTGTTCACTGGGTGCAGAG
**To characterize chimeric transcripts**	
p5516	CGCCCAAGACGGAGACCC
p5518	TCACTGGCTATGTGACGCTG
p5578	TGTGTTCACTGGGTGCAGAG
p5581	AACATCCTCCAACTCGGCAG
p5582	AGAGTTTCCCATCAGGCTGC
P5772	GCATCGGGTAGGGCATCAC
p5670	CACTCGTCAATGATACTGCGC
P5773	TTTTCTTGCCTACGGATCCCC
p5673	TCTGGGTCGTCTTTCACAGC
pJet forward	CGACTCACTATAGGGAGAGCGGC
pJet Reverse	AAGAACATCGATTTTCCATGGCAG
**Primers and probes used for Digital PCR**	
p Forward (ZZEF1 exon 5)	CTATGTGACGCTGCTGGAAAAT
p Reverse (ALOX15 exon 1)	GCGGATGCGGTAGAGACC
Probe (ZZEF1 exon 5)	CCAACGTCAGTCAGC

p Forward (ALOX15 exon 3/4)	TTGTACCGGTGGGGAAACTG
p Reverse (ALOX15 exon 4)	CAGCGAAACCTCAAAGTCAACT
Probe (ALOX15 exon 4)	CCAAACTATATGACCTCCCT
**Primers used to create Chimera/IRES plasmid for animal experiment**	
p5685	AGAATTCCTCGAGATGGGGAACGCTCCGAG TCAC
p5686	AGATCTGCGGCCGCTTAGATGGCCACACTG TTTTC
p5683	TTCAGGAATTCATGGGTCTCTACCGCATCCG
p5684	TTCAGCTCGAGTTAGATGGCCACACTGTTTTCC
Thr560Met_F	ACCCTGCATGATGCGGCTGCCCCCGCCAA
Thr560Met_R	CGCATCATGCAGGGTGCATTAGGCA
**Primers used in UMI-4C Analysis**	
ZZEF1_upDup_US	TTTCTAGAACACTGGTGCTGCA
ZZEF1_upDup_DS	AATGATACGGCGACCACCGAGATCTACACTCTTTCCCTACACGACGCTCTTCCGATCT GGGCTTTCTCTAAAGCCTATCT
ZZEF1_promo_US	GTTGTAATGCAGACCTTTGTGC
ZZEF1_promo_DS	AATGATACGGCGACCACCGAGATCTACACTCTTTCCCTACACGACGCTCTTCCGATCT GCAGTTGATTGACGCTACTC
Alox15_1stIntron_US	CATCCCTTCCCTCTCACCA
Alox15_1stIntron_DS	AATGATACGGCGACCACCGAGATCTACACTCTTTCCCTACACGACGCTCTTCCGATCT CCAGGTGATAAAGCCTGAAAG
Universal Primer	CAAGCAGAAGACGGCATACGA

HB-TruSeq-5	GATCGGAAGAGCACACGTCTGAACTCCAGTCACACAGTGATCTCGTATGCCGTCTTCTGCTTG
HB-TruSeq-12	GATCGGAAGAGCACACGTCTGAACTCCAGTCACCTTGTAATCTCGTATGCCGTCTTCTGCTTG
HB-TruSeq-13	GATCGGAAGAGCACACGTCTGAACTCCAGTCACAGTCAACAATCTCGTATGCCGTCTTCTGCTTG
HB-TruSeq-14	GATCGGAAGAGCACACGTCTGAACTCCAGTCACAGTTCCGTATCTCGTATGCCGTCTTCTGCTTG
HB-TruSeq-15	GATCGGAAGAGCACACGTCTGAACTCCAGTCACATGTCAGAATCTCGTATGCCGTCTTCTGCTTG
HB-TruSeq-16	GATCGGAAGAGCACACGTCTGAACTCCAGTCACCCGTCCCGATCTCGTATGCCGTCTTCTGCTTG
Universal Illumina Adapter	AATGATACGGCGACCACCGAGATCTACACTCTTTCCCTACACGACGCTCTTCCGATC*T

Supplemental Table 6. Number of reads generated per sample from RNA sequencing and the percentage aligned to the hg19 reference genome.

Sample #	Read pairs	% Aligned
III-5 (affected)	46,376,862	95.53%
II-2 (affected)	50,328,599	94.73%
II-4 (affected)	43,029,482	95.80%
III-2 (unaffected)	51,047,868	95.30%
III-4 (unaffected)	48,446,876	93.86%
II-6 (unaffected)	49,693,511	93.32%

Supplemental Table 7: Read counts of the most differentially expressed genes, genome wide based on having both a significant p-value and fold change.

	Affected II-2	Affected III-5	Affected II-4	Unaffected III-2	Unaffected III-4	Unaffected II-6	BH p-value
***ALOX15* (+)**	303.9707	371.0062	408.2963	0.929419	0	2.862046	$3.12E^{-22}$
***TDRD9* (-)**	3.787797	0	3.393044	343.8851	203.6837	59.14894	$1.07E^{-09}$
***IGHV3-48* (-)**	1170.429	554.9416	389.0691	10571.21	6652.682	3757.866	$7.77E^{-09}$
***HLA-H* (-)**	169.5039	125.4105	137.9838	1937.839	1781.002	304.3309	$3.48E^{-05}$
***PILRB* (-)**	75.75594	49.11913	92.74321	183.0956	235.171	213.6994	$9.10E^{-04}$
***LRP1* (-)**	52.08221	50.16422	49.76465	158.9307	118.0775	118.2979	$1.68E^{-03}$
***UNC13A* (-)**	44.50661	35.53299	53.15769	99.44786	154.4847	113.5278	$1.68E^{-03}$
***TRIM47* (+)**	160.0344	156.7632	169.6522	42.75328	56.0868	78.22925	$2.07E^{-03}$
***PLAU* (-)**	89.96018	104.5088	64.46784	240.7196	166.2925	222.2855	$1.68E^{-02}$
***PVRL4* (-)**	77.64984	72.11106	49.76465	131.0481	189.9079	185.0789	$1.68E^{-02}$
***IGDCC4* (-)**	23.67373	19.85667	16.96522	59.48283	56.0868	75.3672	$1.78E^{-02}$
***DRAXIN* (-)**	29.35543	20.90176	21.48928	135.6952	61.99068	77.27523	$1.78E^{-02}$
***IGHV1OR15-1* (-)**	98.48272	87.78738	128.9357	190.5309	259.7705	234.6877	$1.78E^{-02}$
***HCG22* (+)**	213.0636	149.4476	230.727	55.76515	47.23099	100.1716	$2.82E^{-02}$

Genes with a positive symbol are over-expressed in affected compared to unaffected; genes with a negative symbol are under-expressed. Asterisks are used to denote genes that fall within the linkage region.

Supplemental Table 8. Clinical Blood Results of Two Affected Family Members

**Test**	**Proband (III-5; 31 Y, F)**	**Affected Mother (II-4, 67 Y, F)**	**Normal Range**
Haemoglobin	130	137	110 – 151 g/L
RBC count	4.38	4.62	3.70 – 4.87 x 10^{12}/L
Haematocrit	0.389	0.419	0.329 – 0.412 L/L
WBC count	4.82	6.84	4.37 – 9.68 x 10^{9}/L
Platelet count	275	303	186 – 353 x 10^{9}/L
Mean corpuscular volume	88.8	90.7	77.7 – 93.7 fL
Mean corpuscular Haemoglobin	29.7	29.7	25.3 – 30.9 pg
Mean corpuscular Haemoglobin concentration	334	327	310 – 341 g/L
Mean platelet volume	10.3	9.1	7.5 – 11.5 fL
Differential count			
Neutrophil	2.97	5.47	2.00 – 7.15 x 10^{9}/L
Eosinophil	0.04	0.00	0.03 – 0.27 x 19^{9}/L
Basophil	0.00	0.00	0.01 – 0.05 x 10^{9}/L
Lymphocytes	1.43	1.02	1.16 – 3.18 x 10^{9}/L
Monocytes	0.38	0.29	0.29 – 0.71 x 10^{9}/L
Lipid Profile			
Cholesterol	4.79	6.21 (Borderline high)	<5.20 mmol/L
HDL Cholesterol	2.81	2.53	>1.04 mmol/L
LDL Cholesterol	1.65	3.23	<3.50 mmol/L
Triglyceride	0.73	0.99	< 1.70 mmol/L
Iron	18.1	20.4	6.6 – 30.4 µmol/L
Ferritin	37.1	47.4	4.6 – 204.0 µg/L
Transferrin	31.2	35.3	22.6 – 48.0 µmol/L
Liver function tests			
Total protein	69	72	63 – 82 g/l
Albumin	42	45	35 – 50 g/L
ALT	12	19	< 35 U/L
AST	22	30	14 – 36 U/L
GGT	14	24	12 – 43 U/L
Hemoglobin A1C	5	5.6	< 6.0 %
Platelet Aggregation	Normal ATP release. Normal platelet aggregation	Normal ATP release. Normal platelet aggregation	
Liver Ultrasound	No evidence of fatty liver	No evidence of fatty liver	

Supplemental Table 9. ALOX15 Metabolite Disease Associations.

Disease risk	Associated ALOX15 Metabolite	Effect on Disease Risk	Reference
**Asthma/allergies**	Eoxins	Increased	(54)
**Atherogenesis**	15-HETE	Increased	(55, 56)
**Non-alcoholic fatty liver disease**	5-HETE, 8-HETE, 12-HETE, 15-HETE	Increased	(57, 58)
**Type 2 diabetes**	12-HETE, 15-HETE	Increased	(59, 60)
**Stroke**	Lipoxin A4	Decreased	(61-64)
**Periventricular leukomalacia**	12-HETE	Increased	(65, 66)
**Alzheimer's Disease**	12-HETE, 15-HETE	Increased	(67-70)
	Neuroprotectin D1	Decreased	
**Breast cancer**	13-HODE	Increased	(71)
**Prostate cancer**	13-HODE	Increased	(72)
**Multiple sclerosis**	12-HETE	Increased	(73)

Supplemental Table 10. ***ALOX5*** **RNA Read counts.**

	Affected II-2	Affected III-5	Affected II-4	Unaffected III-2	Unaffected III-4	Unaffected II-6	BH p-value
ALOX5	1457.354868	1246.789761	2159.107146	2394.183889	1062.697314	1315.586969	0.94

Supplemental Table 11. List of all viewpoint sequences, and the corresponding bait sequences with the relevant SNPs for UMI-4C analysis.

Bait Viewpoint	Bait Sequence (5’-3’)	Pad Sequence (5’-3’)
ZZEF1_upDup_CTL_C	GGGCTTTCTCTAAAGCCTATCT	AGTACTGACAAAGAAGAAGCTGAGATC
ZZEF1_upDup_MUT_T	GGGCTTTCTCTAAAGCCTATCT	AGTACTGATAAAGAAGAAGCTGAGATC
ZZEF1_promo_ALL	GCAGTTGATTGACGCTACTC	TGGAGATC
Alox15_1stIntron_CTL_T	CCAGGTGATAAAGCCTGAAAG	GTAGAGATC
Alox15_1stIntron_MUT_C	CCAGGTGATAAAGCCTGAAAG	GCAGAGATC

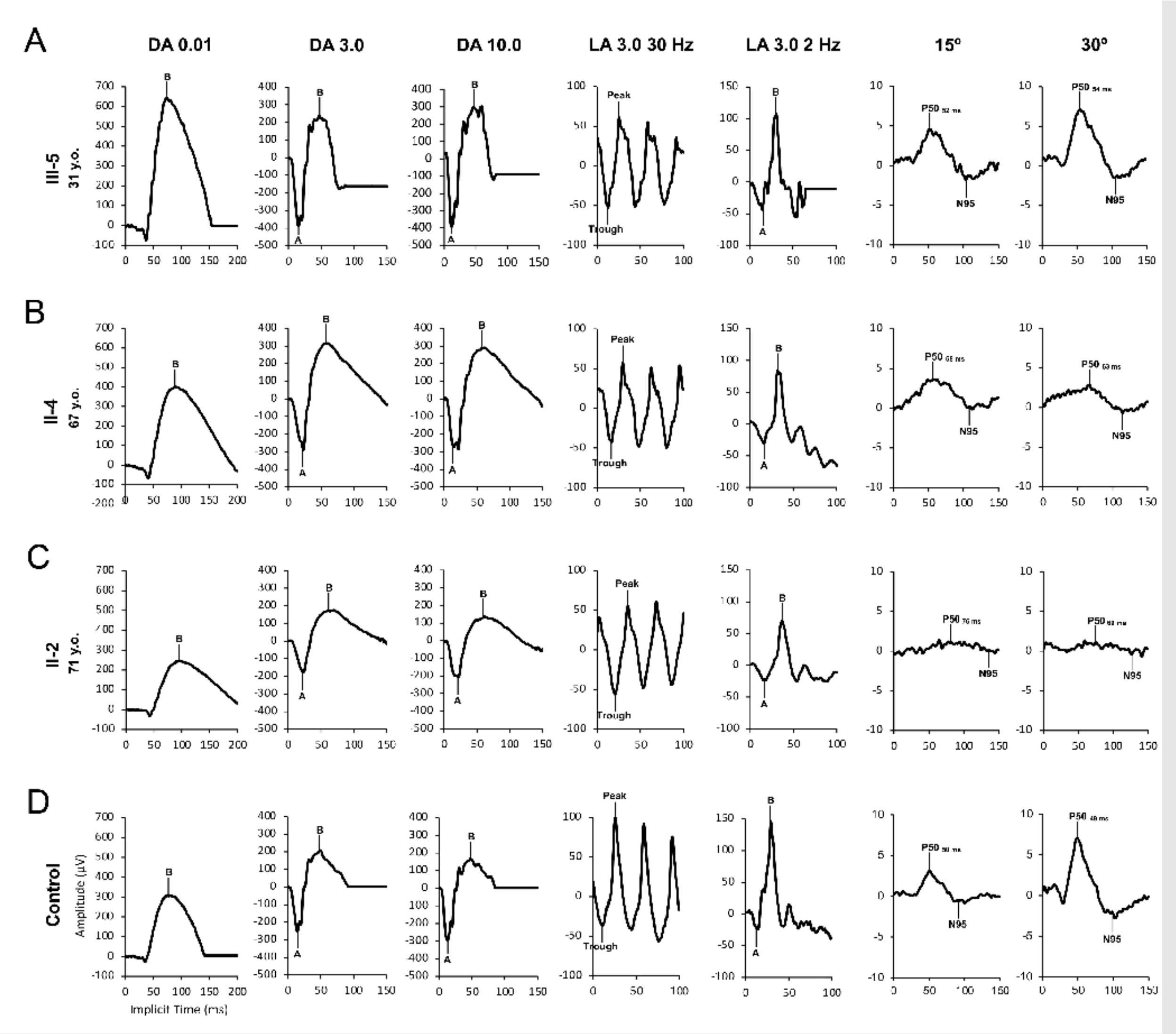

Supplemental Figure 1: Full field electroretinogram (ERG) and Pattern ERG (PERG) findings in three affected individuals. Each panel represents test results from the right eye of one individual. The bottom most panel is a control trace. All steps of the full-field ERG (DA 0.01, DA 3.0, DA 10.0, LA 3.0 30 Hz and LA 3.0 2 Hz) are normal in all three affected individuals (top 3 panels). This excludes any generalized rod and cone system dysfunction in any of the three affected. A standard field (15°) and wide-field (30°) PERG were performed to assess central and peripheral macular function. The proband had normal PERG, where II-4 had peripheral macular dysfunction, whilst II-2 had both central and peripheral macular dysfunction.

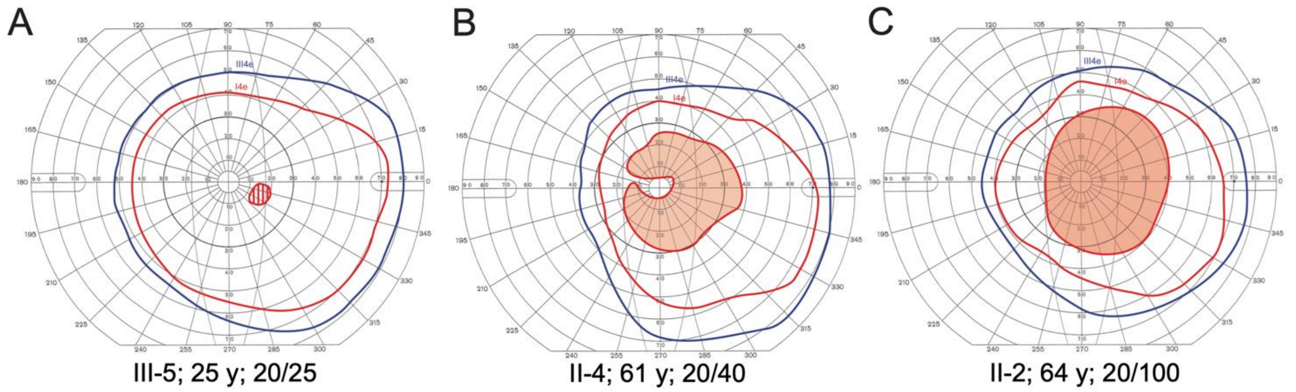

Supplemental Figure 2: Visual field images from affected individuals III-5 (A), II-4 (B) and II-2 (C). The visual fields were normal in III-5 and showed scotoma to the I4e target in II-4 and II-2. The red tracing indicates the I4e target, while the blue indicates III4e. Shading indicates areas of scotoma.

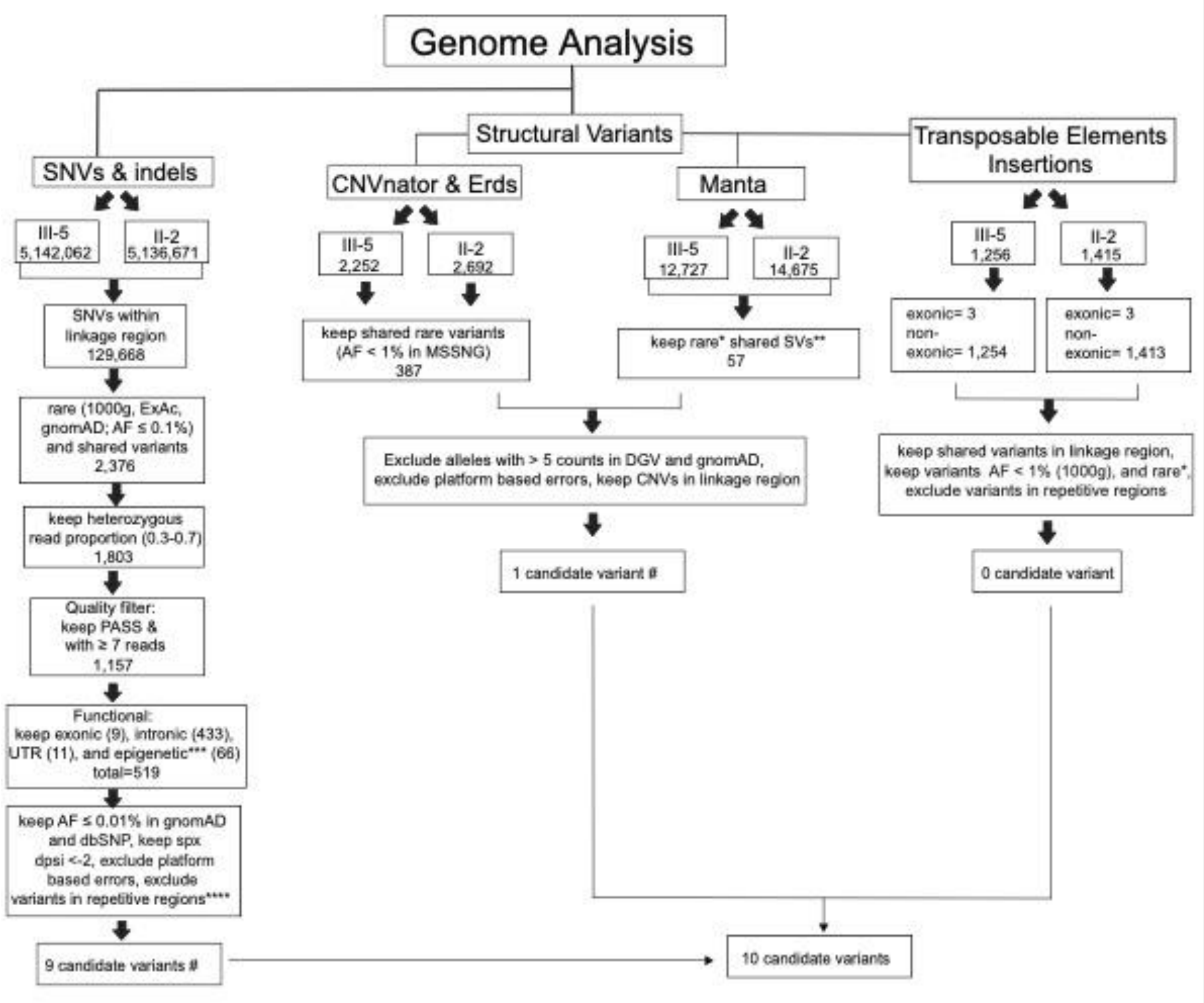

Supplemental Figure 3: Genome filtering pipeline. The schematic shows the filtering pipeline and strategies used to prioritize candidate variants.

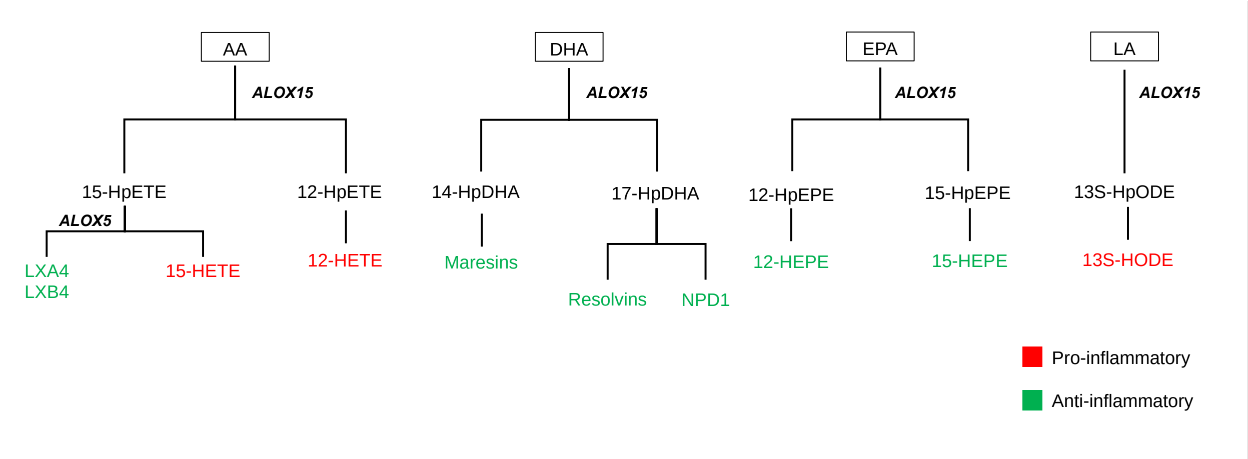

Supplemental Figure 4: ALOX15 pathway. This figure elucidates the various substrates of ALOX15. The substrates include arachidonic acid (AA), docosahexaenoic acid (DHA), eicosapentaenoic acid (EPA), and linoleic acid (LA). The metabolites include 15- and 12-hydroperoxyeicosatetraenoic acid (15- and 12-HpETE), lipoxin A4 and B4 (LXA4/LXB4), 15- and 12-hydroxyeicosatetraenoic acid (15- and 12-HETE), 14- and 17-hydroperoxydocosahexaenoic acid (14- and 17-HpDHA), maresins, resolvins, neuroprotectin D1 (NPD1), 12- and 15-hydroperoxyeicosapentaenoic acid (12- and 15-HpEPE), 12- and 15-hydroxyeicosapentaenoic acid (12- and 15-HEPE), 13(S)-hydroperoxylinoleic acid (13S-HpODE), and 13(S)-hydroxyoctadecadienoic acid (13S-HODE). The metabolites have a wide range of activity and the ones that are pro-inflammatory are labelled in red and the ones that have a protective or anti-inflammatory effect are labelled in green.

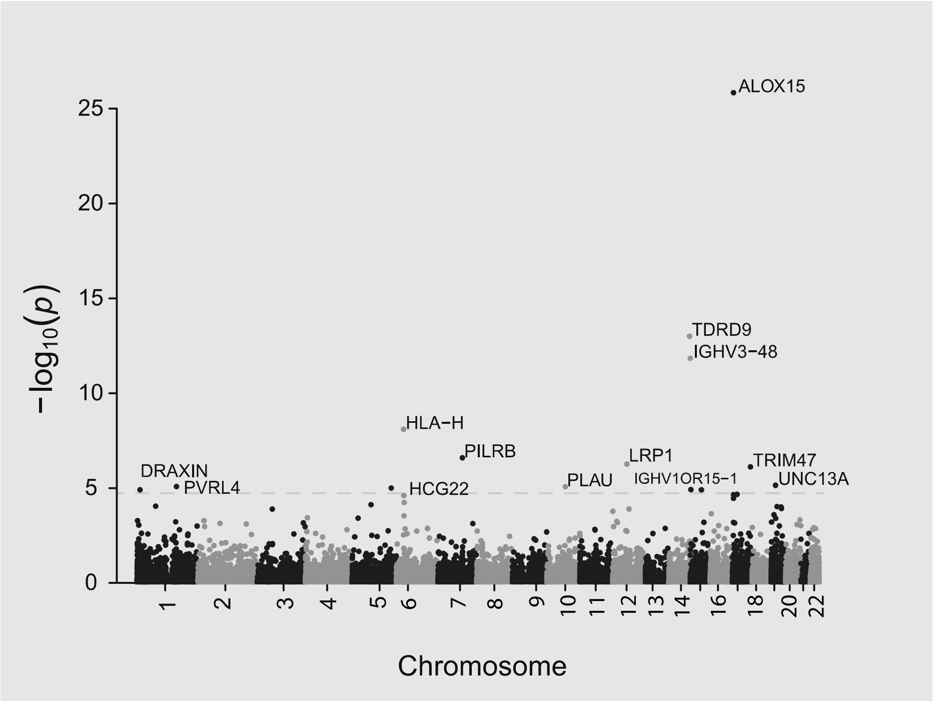

Supplemental Figure 5: Manhattan plot. The plot showed no obvious relationship between genomic position and differential gene expression in chromosome 17 between affected and unaffected individuals. Genes above the dashed line have a significant Benjamini-Hochberg *P*-value ($P \leq 0.00001$).

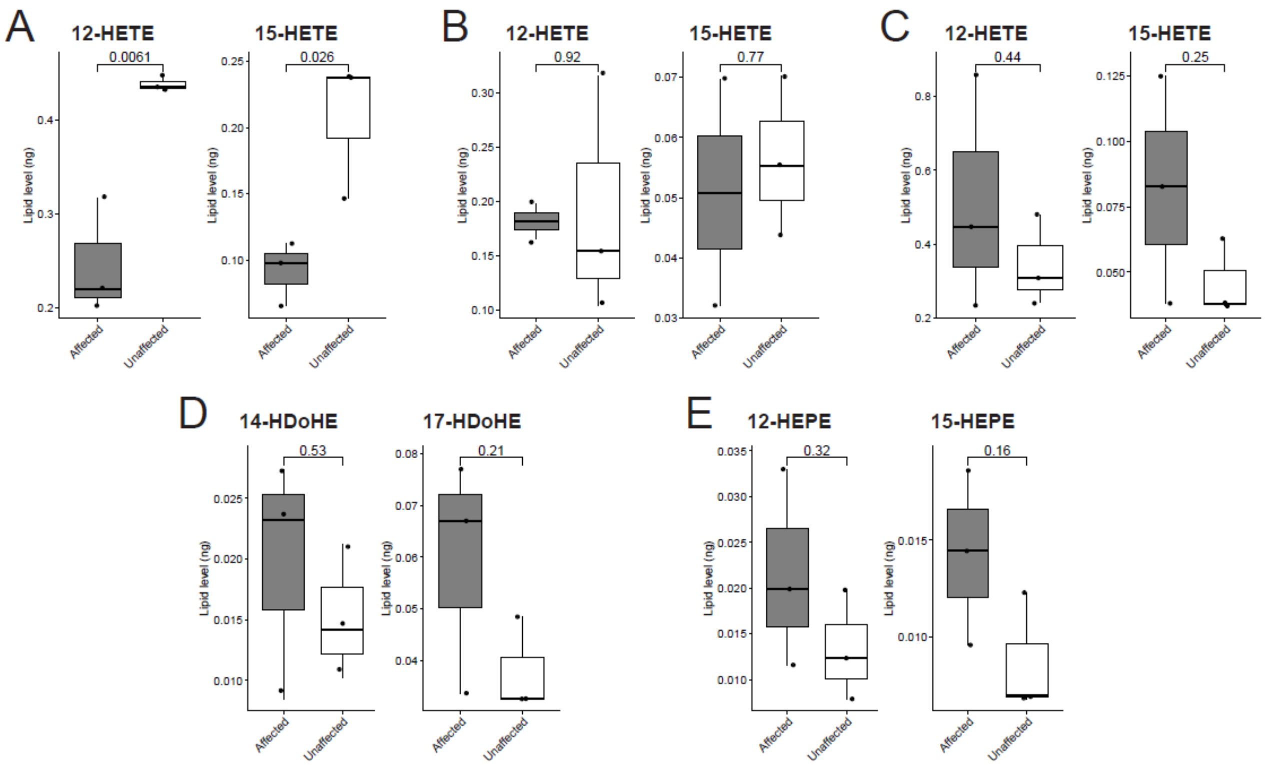

Supplemental Figure 6: Liquid Chromaytography Mass Spectrometry (LC-MS) results of ALOX15 metabolites in affected (n=3) and unaffected (n=3) lymphoblast cell lines (LCLs). To examine for arachidonic acid derived metabolite levels (15- and 12-HETE), three separate experiments using 18-20 million cells (in triplicates) were done (A-C); the levels of 15- and 12-HETE were low in both patients and controls, and variable, and hence, the results are considered unreliable for further interpretation (two tailed, unpaired t-test). (D) Analysis of DHA-derived metabolites (20 million cells in triplicates) 17-HDoHE and 14-HDoHE were also at the lower level of detection of LCMS in both patients and controls (two tailed, unpaired t-test). (E) Analysis of EPA-derived metabolites 12- and 15-HEPE (20 million cells in triplicates) were also low in patients and controls (two tailed, unpaired t-test). Note: The lower end of the LCMS detection curve is 0.01 ng.

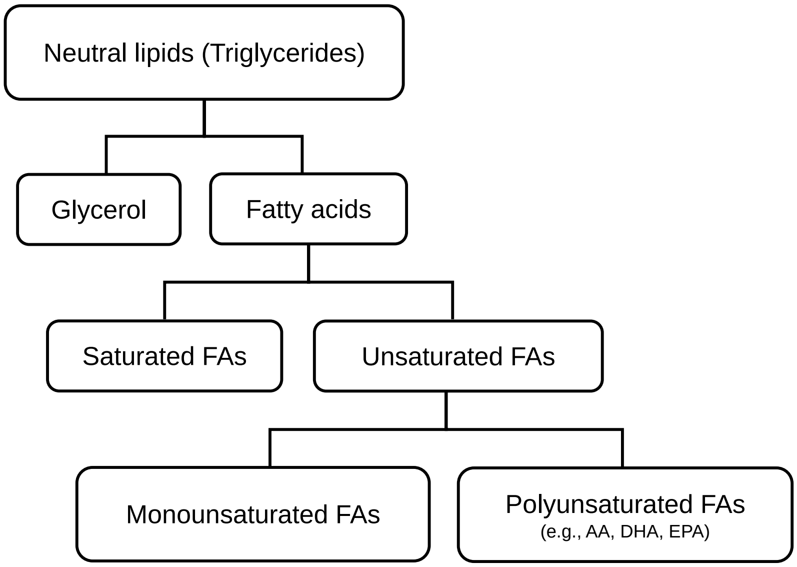

Supplemental Figure 7: Neutral lipids composition. Triglycerides are composed of glycerol backbones connected to fatty acid (FA) tails. One subset of these FAs are PUFAs, including AA, DHA, and EPA, the levels of which depend on the tissue examined. For example, PUFAs make up about 27-37% of FAs in serum (74-76).

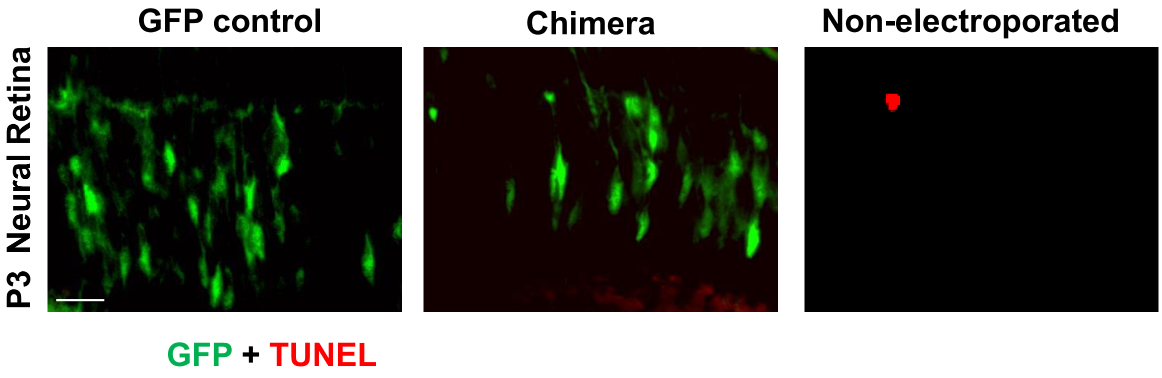

Supplemental Figure 8: Electroporation with Chimera does not cause early retinal photoreceptor death in mouse retinas. P0 C57BL/6J mice were given subretinal injections of pT2K-IRES-eGFP (GFP control) or pT2K-chimera-IRES-eGFP (Chimera 1), followed by electroporation of developing photoreceptors. Eyes were harvested 3 days later (n = 3 each in GFP control and Chimera 1 group, >3 sections in each eye), 9um thick cryosections from the central retinal were generated and terminal deoxynucleotidyl transferase-mediated dUTP nick end labeling (TUNEL) (red) staining was performed. Electroporated cells in neural retina are labelled in green. The RPE (not shown) is located above the neural retina and the vitreous (not shown) is located below. TUNEL positive cells could be sparsely observed in non-electroporated regions of the retina, representing natural developmental cell death (right). Electroporation with GFP control (left) or Chimera (middle) were not associated with any increases in the number of TUNEL positive cells at P3. The faint red signal seen in middle image, is background noise from streptavidin. True positive cells will have a strong signal from the biotin labelling and distinct nuclei shape. Scale bar 20 µm.

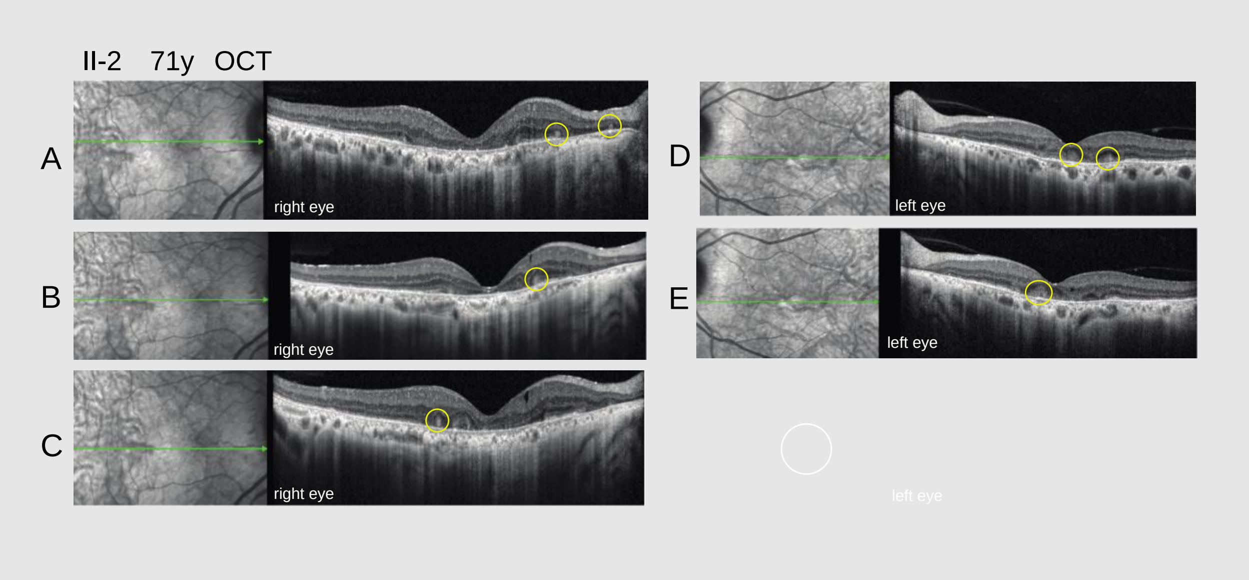

Supplemental Figure 9: Optical coherence tomography (OCT) of individual II-2. (A-C) are images form the right eye while (D) and (E) are images from the left eye. The OCT shows iso-reflective rounded structures representing rosettes (highlighted with circles) in all these scans from within the macula. Additionally, there is marked disruption of outer retinal lamination including disruption of the ellipsoid zone, external limiting membrane and outer nuclear layer; as well as thinning of the retinal pigment epithelium.

Supplemental References

1. Purcell S, Neale B, Todd-Brown K, Thomas L, Ferreira MA, Bender D, et al. PLINK: a tool set for whole-genome association and population-based linkage analyses. *Am J Hum Genet.* 2007;81(3):559-75.
2. Gazal S, Sahbatou M, Babron M-C, Génin E, and Leutenegger A-L. FSuite: exploiting inbreeding in dense SNP chip and exome data. *Bioinformatics.* 2014;30(13):1940-1.
3. Wigginton JE, and Abecasis GR. PEDSTATS: descriptive statistics, graphics and quality assessment for gene mapping data. *Bioinformatics.* 2005;21(16):3445-7.
4. Schaffer AA, Lemire M, Ott J, Lathrop GM, and Weeks DE. Coordinated conditional simulation with SLINK and SUP of many markers linked or associated to a trait in large pedigrees. *Hum Hered.* 2011;71(2):126-34.
5. Abecasis GR, Cherny SS, Cookson WO, and Cardon LR. Merlin--rapid analysis of dense genetic maps using sparse gene flow trees. *Nat Genet.* 2002;30(1):97-101.
6. Auton A, Brooks LD, Durbin RM, Garrison EP, Kang HM, Korbel JO, et al. A global reference for human genetic variation. *Nature.* 2015;526(7571):68-74.
7. Lionel AC, Costain G, Monfared N, Walker S, Reuter MS, Hosseini SM, et al. Improved diagnostic yield compared with targeted gene sequencing panels suggests a role for whole-genome sequencing as a first-tier genetic test. *Genet Med.* 2018;20(4):435-43.
8. Andrews S. FastQC: a quality control tool for high throughput sequence data. 2010.
9. Roller E, Ivakhno S, Lee S, Royce T, and Tanner S. Canvas: versatile and scalable detection of copy number variants. *Bioinformatics.* 2016;32(15):2375-7.
10. Chen X, Schulz-Trieglaff O, Shaw R, Barnes B, Schlesinger F, Kallberg M, et al. Manta: rapid detection of structural variants and indels for germline and cancer sequencing applications. *Bioinformatics.* 2016;32(8):1220-2.
11. Abyzov A, Urban AE, Snyder M, and Gerstein M. CNVnator: an approach to discover, genotype, and characterize typical and atypical CNVs from family and population genome sequencing. *Genome Res.* 2011;21(6):974-84.
12. Zhu M, Need AC, Han Y, Ge D, Maia JM, Zhu Q, et al. Using ERDS to infer copy-number variants in high-coverage genomes. *Am J Hum Genet.* 2012;91(3):408-21.
13. Thung DT, de Ligt J, Vissers LE, Steehouwer M, Kroon M, de Vries P, et al. Mobster: accurate detection of mobile element insertions in next generation sequencing data. *Genome Biol.* 2014;15(10):488.
14. Wang K, Li M, and Hakonarson H. ANNOVAR: functional annotation of genetic variants from high-throughput sequencing data. *Nucleic Acids Res.* 2010;38(16):e164.
15. Lek M, Karczewski KJ, Minikel EV, Samocha KE, Banks E, Fennell T, et al. Analysis of protein-coding genetic variation in 60,706 humans. *Nature.* 2016;536(7616):285-91.
16. Karczewski KJ, Francioli LC, Tiao G, Cummings BB, Alfoldi J, Wang Q, et al. The mutational constraint spectrum quantified from variation in 141,456 humans. *Nature.* 2020;581(7809):434-43.
17. Yuen RKC, Merico D, Bookman M, Howe JL, Thiruvahindrapuram B, Patel RV, et al. Whole genome sequencing resource identifies 18 new candidate genes for autism spectrum disorder. *Nat Neurosci.* 2017a;20(4):602-11.
18. Tanna P, Strauss RW, Fujinami K, and Michaelides M. Stargardt disease: clinical features, molecular genetics, animal models and therapeutic options. *British Journal of Ophthalmology.* 2017;101(1):25-30.

19. Rahman N, Georgiou M, Khan KN, and Michaelides M. Macular dystrophies: clinical and imaging features, molecular genetics and therapeutic options. *British Journal of Ophthalmology.* 2020;104(4):451-60.
20. Maurano MT, Haugen E, Sandstrom R, Vierstra J, Shafer A, Kaul R, et al. Large-scale identification of sequence variants impacting human transcription factor occupancy in vivo. *Nature genetics.* 2015;47(12):1393-401.
21. Radeke MJ, Radeke CM, Shih Y-H, Hu J, Bok D, Johnson LV, et al. Restoration of mesenchymal retinal pigmented epithelial cells by TGFβ pathway inhibitors: implications for age-related macular degeneration. *Genome Med.* 2015;7(1).
22. Sandström J, Heiduschka P, Beck SC, Philippar U, Seeliger MW, Schraermeyer U, et al. Degeneration of the mouse retina upon dysregulated activity of serum response factor. *Mol Vis.* 2011;17:1110-27.
23. Xiong HY, Alipanahi B, Lee LJ, Bretschneider H, Merico D, Yuen RKC, et al. RNA splicing. The human splicing code reveals new insights into the genetic determinants of disease. *Science.* 2015;347(6218):1254806.
24. Adzhubei IA, Schmidt S, Peshkin L, Ramensky VE, Gerasimova A, Bork P, et al. A method and server for predicting damaging missense mutations. *Nat Methods.* 2010;7(4):248-9.
25. Kumar P, Henikoff S, and Ng PC. Predicting the effects of coding non-synonymous variants on protein function using the SIFT algorithm. *Nat Protoc.* 2009;4(7):1073-81.
26. Choi Y, and Chan AP. PROVEAN web server: a tool to predict the functional effect of amino acid substitutions and indels. *Bioinformatics.* 2015;31(16):2745-7.
27. Kircher M, Witten DM, Jain P, O'Roak BJ, Cooper GM, and Shendure J. A general framework for estimating the relative pathogenicity of human genetic variants. *Nature Genetics.* 2014;46(3):310-5.
28. Reva B, Antipin Y, and Sander C. Predicting the functional impact of protein mutations: application to cancer genomics. *Nucleic Acids Research.* 2011;39(17):e118.
29. Pollard KS, Hubisz MJ, Rosenbloom KR, and Siepel A. Detection of nonneutral substitution rates on mammalian phylogenies. *Genome Research.* 2010;20(1):110-21.
30. Jaganathan K, Kyriazopoulou Panagiotopoulou S, McRae JF, Darbandi SF, Knowles D, Li YI, et al. Predicting Splicing from Primary Sequence with Deep Learning. *Cell.* 2019;176(3):535-48.e24.
31. InteractiveBiosoftware. Alamut mutation interpretation software (Interactive Biosoftware, Rouen, France)
32. Shapiro MB, and Senapathy P. RNA splice junctions of different classes of eukaryotes: sequence statistics and functional implications in gene expression. *Nucleic Acids Res.* 1987;15(17):7155-74.
33. Yeo G, and Burge CB. Maximum entropy modeling of short sequence motifs with applications to RNA splicing signals. *J Comput Biol.* 2004;11(2-3):377-94.
34. Pertea M, Lin X, and Salzberg SL. GeneSplicer: a new computational method for splice site prediction. *Nucleic Acids Res.* 2001;29(5):1185-90.
35. Reese MG, Eeckman FH, Kulp D, and Haussler D. Improved splice site detection in Genie. *Journal of Computational Biology: A Journal of Computational Molecular Cell Biology.* 1997;4(3):311-23.
36. Desmet F-O, Hamroun D, Lalande M, Collod-Béroud G, Claustres M, and Béroud C. Human Splicing Finder: an online bioinformatics tool to predict splicing signals. *Nucleic Acids Research.* 2009;37(9):e67-e.
37. Robinson JT, Thorvaldsdóttir H, Winckler W, Guttman M, Lander ES, Getz G, et al. Integrative Genomics Viewer. *Nat Biotechnol.* 2011;29(1):24-6.
38. Kent WJ. BLAT--the BLAST-like alignment tool. *Genome Res.* 2002;12(4):656-64.

39. Kearse M, Moir R, Wilson A, Stones-Havas S, Cheung M, Sturrock S, et al. Geneious Basic: an integrated and extendable desktop software platform for the organization and analysis of sequence data. *Bioinformatics.* 2012;28(12):1647-9.
40. Bolger AM, Lohse M, and Usadel B. Trimmomatic: a flexible trimmer for Illumina sequence data. *Bioinformatics (Oxford, England).* 2014;30(15):2114-20.
41. Church DM, Schneider VA, Graves T, Auger K, Cunningham F, Bouk N, et al. Modernizing reference genome assemblies. *PLoS Biol.* 2011;9(7):e1001091.
42. Dobin A, Davis CA, Schlesinger F, Drenkow J, Zaleski C, Jha S, et al. STAR: ultrafast universal RNA-seq aligner. *Bioinformatics (Oxford, England).* 2013;29(1):15-21.
43. Li H, Handsaker B, Wysoker A, Fennell T, Ruan J, Homer N, et al. The Sequence Alignment/Map format and SAMtools. *Bioinformatics.* 2009;25(16):2078-9.
44. Banerjee P, Harada H, Tassew NG, Charish J, Goldschneider D, Wallace VA, et al. Upsilon-secretase and LARG mediate distinct RGMa activities to control appropriate layer targeting within the optic tectum. *Cell Death Differ.* 2016;23(3):442-53.
45. Urasaki A, Morvan G, and Kawakami K. Functional dissection of the Tol2 transposable element identified the minimal cis-sequence and a highly repetitive sequence in the subterminal region essential for transposition. *Genetics.* 2006;174(2):639-49.
46. Matsuda T, and Cepko CL. Electroporation and RNA interference in the rodent retina in vivo and in vitro. *Proc Natl Acad Sci U S A.* 2004;101(1):16-22.
47. de Melo J, and Blackshaw S. In vivo Electroporation of Developing Mouse Retina. *J Vis Exp.* 2011(52).
48. Charish J, Shabanzadeh AP, Chen D, Mehlen P, Sethuramanujam S, Harada H, et al. Neogenin neutralization prevents photoreceptor loss in inherited retinal degeneration. *J Clin Invest.* 2020;130(4):2054-68.
49. Tabbarah S, Tavares E, Charish J, Vincent A, Paterson A, Di Scipio M, et al. COG5 variants lead to complex early onset retinal degeneration, upregulation of PERK and DNA damage. *Sci Rep.* 2020;10.
50. Nickerson JM, Goodman P, Chrenek MA, Bernal CJ, Berglin L, Redmond TM, et al. Subretinal delivery and electroporation in pigmented and nonpigmented adult mouse eyes. *Methods Mol Biol.* 2012;884:53-69.
51. Bodenstein L, and Sidman RL. Growth and development of the mouse retinal pigment epithelium. I. Cell and tissue morphometrics and topography of mitotic activity. *Dev Biol.* 1987;121(1):192-204.
52. Schwartzman O, Mukamel Z, Oded-Elkayam N, Olivares-Chauvet P, Lubling Y, Landan G, et al. UMI-4C for quantitative and targeted chromosomal contact profiling. *Nat Methods.* 2016;13(8):685-91.
53. Ramos-Rodriguez M, Subirana-Granes M, and Pasquali L. UMI4Cats: an R package to analyze chromatin contact profiles obtained by UMI-4C. *Bioinformatics.* 2021;37(22):4240-2.
54. Feltenmark S, Gautam N, Brunnstrom A, Griffiths W, Backman L, Edenius C, et al. Eoxins are proinflammatory arachidonic acid metabolites produced via the 15-lipoxygenase-1 pathway in human eosinophils and mast cells. *Proc Natl Acad Sci U S A.* 2008;105(2):680-5.
55. Bajpai AK, Blaskova E, Pakala SB, Zhao T, Glasgow WC, Penn JS, et al. 15(S)-HETE production in human retinal microvascular endothelial cells by hypoxia: Novel role for MEK1 in 15(S)-HETE induced angiogenesis. *Invest Ophthalmol Vis Sci.* 2007;48(11):4930-8.
56. Harats D, Shaish A, George J, Mulkins M, Kurihara H, Levkovitz H, et al. Overexpression of 15-lipoxygenase in vascular endothelium accelerates early atherosclerosis in LDL receptor-deficient mice. *Arterioscler Thromb Vasc Biol.* 2000;20(9):2100-5.

57. Martinez-Clemente M, Ferre N, Titos E, Horrillo R, Gonzalez-Periz A, Moran-Salvador E, et al. Disruption of the 12/15-lipoxygenase gene (Alox15) protects hyperlipidemic mice from nonalcoholic fatty liver disease. *Hepatology.* 2010;52(6):1980-91.
58. Puri P, Wiest MM, Cheung O, Mirshahi F, Sargeant C, Min HK, et al. The plasma lipidomic signature of nonalcoholic steatohepatitis. *Hepatology.* 2009;50(6):1827-38.
59. Dobrian AD, Huyck RW, Glenn L, Gottipati V, Haynes BA, Hansson GI, et al. Activation of the 12/15 lipoxygenase pathway accompanies metabolic decline in db/db pre-diabetic mice. *Prostaglandins Other Lipid Mediat.* 2018;136:23-32.
60. Hatley ME, Srinivasan S, Reilly KB, Bolick DT, and Hedrick CC. Increased production of 12/15 lipoxygenase eicosanoids accelerates monocyte/endothelial interactions in diabetic db/db mice. *J Biol Chem.* 2003;278(28):25369-75.
61. Ivanov I, Kuhn H, and Heydeck D. Structural and functional biology of arachidonic acid 15-lipoxygenase-1 (ALOX15). *Gene.* 2015;573(1):1-32.
62. Jin G, Arai K, Murata Y, Wang S, Stins MF, Lo EH, et al. Protecting against cerebrovascular injury: contributions of 12/15-lipoxygenase to edema formation after transient focal ischemia. *Stroke.* 2008;39(9):2538-43.
63. Pallast S, Arai K, Pekcec A, Yigitkanli K, Yu Z, Wang X, et al. Increased nuclear apoptosis-inducing factor after transient focal ischemia: a 12/15-lipoxygenase-dependent organelle damage pathway. *J Cereb Blood Flow Metab.* 2010;30(6):1157-67.
64. van Leyen K, Kim HY, Lee SR, Jin G, Arai K, and Lo EH. Baicalein and 12/15-lipoxygenase in the ischemic brain. *Stroke.* 2006;37(12):3014-8.
65. Haynes RL, and van Leyen K. 12/15-lipoxygenase expression is increased in oligodendrocytes and microglia of periventricular leukomalacia. *Dev Neurosci.* 2013;35(2-3):140-54.
66. Wang H, Li J, Follett PL, Zhang Y, Cotanche DA, Jensen FE, et al. 12-Lipoxygenase plays a key role in cell death caused by glutathione depletion and arachidonic acid in rat oligodendrocytes. *Eur J Neurosci.* 2004;20(8):2049-58.
67. Lukiw WJ, Cui JG, Marcheselli VL, Bodker M, Botkjaer A, Gotlinger K, et al. A role for docosahexaenoic acid-derived neuroprotectin D1 in neural cell survival and Alzheimer disease. *J Clin Invest.* 2005;115(10):2774-83.
68. Pratico D, Zhukareva V, Yao Y, Uryu K, Funk CD, Lawson JA, et al. 12/15-lipoxygenase is increased in Alzheimer's disease: possible involvement in brain oxidative stress. *Am J Pathol.* 2004;164(5):1655-62.
69. Yang H, Zhuo JM, Chu J, Chinnici C, and Pratico D. Amelioration of the Alzheimer's disease phenotype by absence of 12/15-lipoxygenase. *Biol Psychiatry.* 2010;68(10):922-9.
70. Yao Y, Clark CM, Trojanowski JQ, Lee VM, and Pratico D. Elevation of 12/15 lipoxygenase products in AD and mild cognitive impairment. *Ann Neurol.* 2005;58(4):623-6.
71. O'Flaherty JT, Wooten RE, Samuel MP, Thomas MJ, Levine EA, Case LD, et al. Fatty acid metabolites in rapidly proliferating breast cancer. *PLoS One.* 2013;8(5):e63076.
72. Kelavkar UP, Nixon JB, Cohen C, Dillehay D, Eling TE, and Badr KF. Overexpression of 15-lipoxygenase-1 in PC-3 human prostate cancer cells increases tumorigenesis. *Carcinogenesis.* 2001;22(11):1765-73.
73. Xu J, Zhang Y, Xiao Y, Ma S, Liu Q, Dang S, et al. Inhibition of 12/15-lipoxygenase by baicalein induces microglia PPARbeta/delta: a potential therapeutic role for CNS autoimmune disease. *Cell Death Dis.* 2013;4(4):e569.
74. Zeleniuch-Jacquotte A, Chajes V, Van Kappel AL, Riboli E, and Toniolo P. Reliability of fatty acid composition in human serum phospholipids. *Eur J Clin Nutr.* 2000;54(5):367-72.

75. Sledzinski T, Mika A, Stepnowski P, Proczko-Markuszewska M, Kaska L, Stefaniak T, et al. Identification of cyclopropaneoctanoic acid 2-hexyl in human adipose tissue and serum. *Lipids.* 2013;48(8):839-48.

76. Jannasch F, Bedu-Addo G, Schulze MB, Mockenhaupt FP, and Danquah I. Serum phospholipid fatty acids, dietary patterns and type 2 diabetes among urban Ghanaians. *Nutr J.* 2017;16(1):63.
