# Supplementary material for: Autosomal-dominant macular dystrophy linked to a chromosome 17 tandem duplication
Source: JCI Insight. 2024 Dec 6;9(23):e178768. doi: 10.1172/jci.insight.178768 (PMC11623951; doi:10.1172/jci.insight.178768)
Supplement: Unedited blot and gel images [file jciinsight-9-178768-s094.pdf]

Full unedited gel for Figure 3B

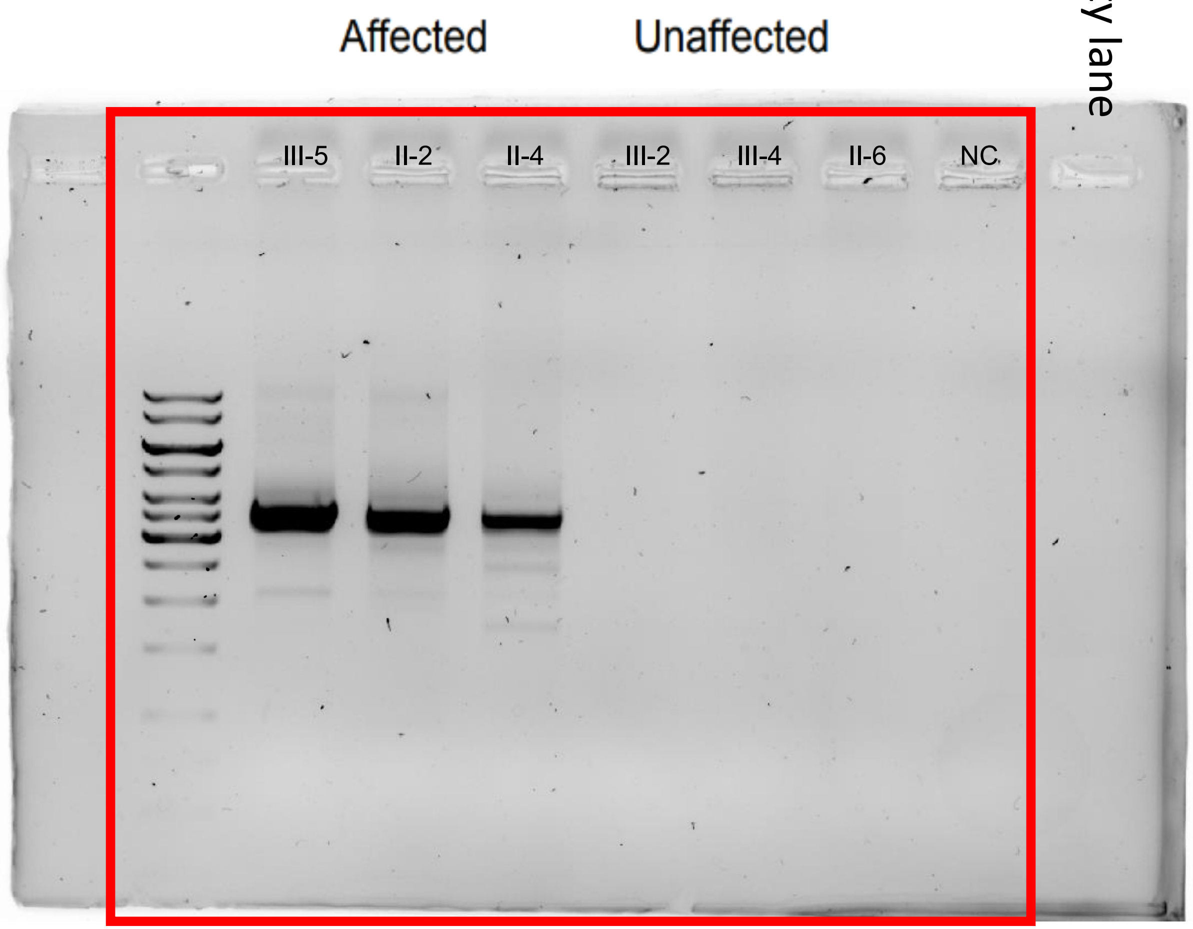

Gel electrophoresis: Only affected individuals show bands of similar size to the predicted model for the chimera 1 transcript. NC =negative control.

Full unedited blot for Figure 5A

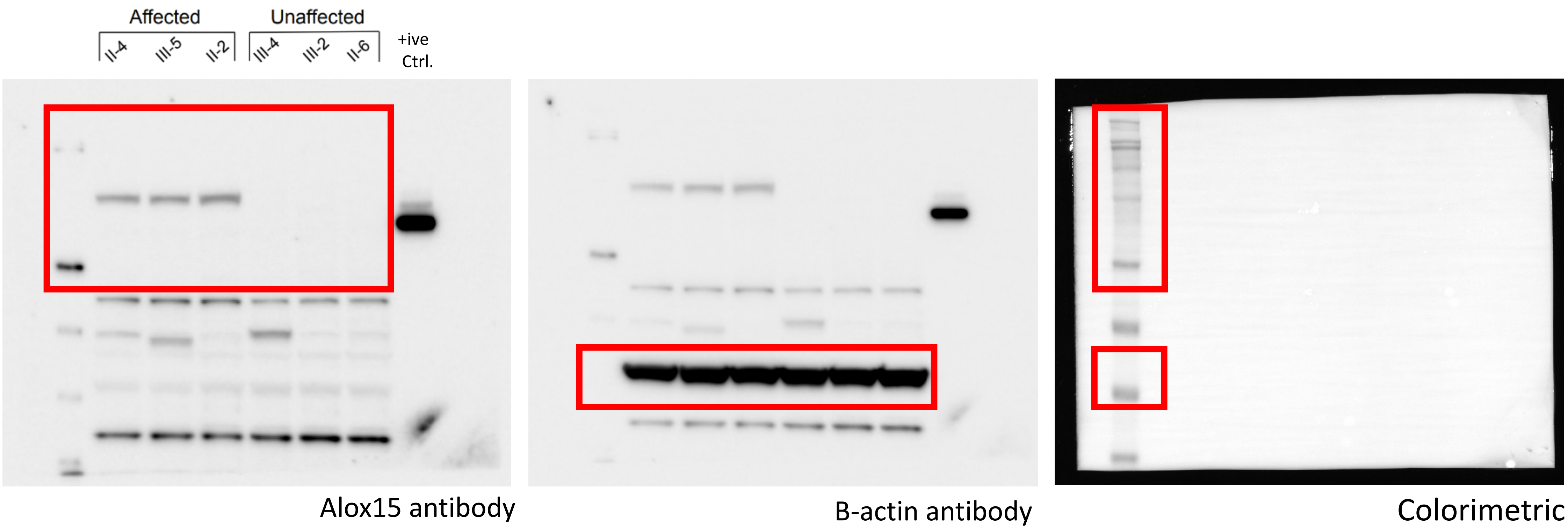

The left panel shows the blot after labelling with the Alox15 antibody. In the last lane, 0.75ng of ALOX15 protein (Abcam, ab114421) was loaded as a positive control for the antibody.

The middle panel shows the same blot after labelling with B-actin antibody (Sigma-Aldrich A1978).

The panel on the right is the colorimetric image of the blot to show the position of the HiMark Pre-stained Protein Standard (Thermo Fisher Scientific).
